# Screening and characterization of soil bacteria for lignin and textile dye effluent bioremediation and optimization using response surface methodology

**DOI:** 10.1038/s41598-025-04789-5

**Published:** 2025-07-16

**Authors:** Sarah Milad, Sarra E. Saleh, Mohammad M. Aboulwafa, Nadia A. Hassouna

**Affiliations:** 1 Department of Microbiology and Immunology, Faculty of Pharmacy, Ain Shams University, Cairo, Egypt; 2 Faculty of Pharmacy, King Salman International University, Ras Sudr, South Sinai Egypt

**Keywords:** Soil bacteria, Kraft lignin, Ligninolytic enzymes, Azure B, Dye decolorization, Biotechnology, Microbiology

## Abstract

In this study, 177 bacterial isolates were recovered from 55 agricultural soil samples collected from various locations in Egypt. Following purification, the isolates were evaluated in solid and liquid phase assays for their capacity to decolorize several types of dyes such as Azure B (AB), methylene blue (MB), and Congo red (CR). The 16S rRNA sequence analysis was used to identify isolates with the highest decolorizing capacity. The two bacterial isolates coded 304 and 434 which exhibited potential ligninolytic activity were identified as *Streptomyces griseorubens* and *Streptomyces intermedius*, respectively. *Streptomyces intermedius* test isolate was selected for optimization experiments using the one-factor-at-a-time approach (OFAT) followed by a statistical method of optimization using response surface methodology (RSM). The optimization experiments resulted in a 2.6-fold increase in dye decolorization capacity after 4 h of incubation with bacterium growth compared to basal conditions and thus indicated a significant reduction in dye decolorization time, accelerating the dye decolorization process and demonstrating enhanced efficiency in ligninolytic enzyme production. In addition, the whole genome sequencing (WGS) process was performed on *S. intermedius* isolate to detect the relevant genes related to lignin degradation and dye decolorizing activities. After annotation and analysis of the genomic sequence, various genes encoding enzymes related to lignin degradation and dye decolorization activities were identified confirming the genetic potential of this strain for efficient ligninolytic activity. The obtained WGS data was deposited in the NCBI database under the accession code SRR25321249. Taken together, the WGS data are in alignment with phenotypic dye decolorization activity of the selected isolate. Accordingly, the test isolate *S. intermedius* 434 was considered a potential candidate for lignin biodegradation and textile dye effluent bioremediation.

## Introduction

Lignin is considered one of the most prevalent polymers on earth. It naturally exists in plants as an integral part of lignocellulose to give them structure and rigidity^1^. Most technical lignins available today are byproducts of pulping processes, primarily kraft, soda, and sulfite methods^2^. Among them, the kraft process is the industry standard for paper and pulp production on a global scale. Each year, 130 million tons of kraft pulp are produced which may cause serious pollution to aquatic ecosystems upon direct disposal^3^. This abundant polymer can be broken down and transformed into molecules with higher value, allowing us to extract even more valuable products from it^4^.

Lignin degradation can be accomplished by biological processes using entire cells (bacterial or fungal biomass) or ligninolytic enzymes^5^, offering an energy efficient, environmentally safe, and selective method^6^. While white-rot fungi produce powerful lignin degrading enzymes, their industrial applicability is limited^7^. In contrast, bacteria tolerate broader conditions and are easier for genetic modifications^8,9^.

Laccase, lignin peroxidase (LiP), manganese peroxidase (MnP), versatile peroxidase (VP), and dye-decolorizing peroxidase (DyP) are examples of ligninolytic enzymes that can be produced by fungi and bacteria found in plant biomass and soil^10,11^. In biochemical research on ligninolytic bacteria, LiPs, MnPs, and VPs have not been observed. No homologs are discovered even when sequencing ligninolytic bacteria’s genomes or proteomes, implying fungi as their only known source. It has lately become obvious that DyPs, a different class of peroxidase, are quite abundant in bacteria. Although DyPs are structurally unrelated to the common fungal peroxidases, they do share several catalytic characteristics as well as redox potentials and reactivities^12^.

The use of bacterial ligninolytic enzyme systems has been expanding rapidly over the last few decades due to their potential in industrial processes and environmental applications^7,13^. Recent research has focused on separating different ligninolytic bacteria for usage in a range of biotechnological processes, including bioremediation^14^, biofuel production^15^, and industrial enzyme development^16^. The ligninolytic enzymes hold great promise for industrial use in a variety of fields, such as food processing, cosmetics, aromatic compounds degradation, lignin valorization, and dye effluent decolorization^17,18^.

The textile industry uses thousands of synthetic dyes to make fabrics. Over one-third of these are not absorbed by the fabric and end up in wastewater, threatening aquatic biodiversity and human health due to their toxicity and carcinogenicity^19^. Although physicochemical treatment techniques have been employed to eliminate dyes, they are not ecologically friendly nor commercially practical. On the other hand, biological methods are cheaper, effective, and sustainable for the environment^20^.

Laccases and peroxidases, two ligninolytic enzymes, efficiently remove dyes, helping reduce textile-related water pollution^21^. Dye biodegradation entails intricate biochemical reactions that break down dye molecules into simpler, more biodegradable, and non-toxic byproducts^20,22^. Decolorization occurs when the dyes lose their chromophores as a result of repeated oxidation processes ^23^.

Recent approaches emphasize the use of microbes or their enzymes to effectively treat textile dye effluents, incorporating different immobilization methods and bioreactor designs tailored for systems utilizing immobilized agents^24^. There are many reports on ligninolytic bacteria and their applications in dye degradation such as decolorization of methylene blue dye by a ligninolytic enzyme-producing *Bacillus thuringiensis* ^25^, multiple dye degradation by a newly isolated ligninolytic bacteria *Bacillus paramycoides*^21^*,* and decolorization of textile azo dyes by *Streptomyces albidoflavus* 3MGH^26^.

Several studies have focused on optimizing the decolorization of various dyes, including Azure B (AB), methylene blue (MB), and Congo red (CR). *Serratia liquefaciens*, a lignin peroxidase-producing bacterium, demonstrated rapid decolorization of AB dye (100 mg/L) in mineral salt medium supplemented with glucose and yeast extract, achieving over 90% decolorization within 48 h^27^. Similarly, *Bacillus sp*. MZS10 achieved 93.55% decolorization of AB dye within 14 h in a stirred-tank fermenter, with the process being dependent on cell density and optimized medium conditions^28^. Regarding MB, thermophilic *Bacillus licheniformis* B3-15 and *Bacillus* sp. s7s-1 showed the ability to decolorize and detoxify MB dye in aqueous solution using preformed biofilms on polypropylene perforated balls (BBs). At optimized initial conditions (10 mg L^−1^ MB, pH 5.2 for B3-15 or pH 4 for s7s-1), the two strains enhanced their decolorization potential, reaching 96 and 67%, respectively, showing significant detoxification potential^29^. In case of CR, *Alcaligenes faecalis* H77, bacterium isolated from textile wastewater, proved to be highly effective in biodegrading CR. Through optimization process, it decolorized 92.5% of CR (25 mg/L) in 72 h at pH 6.7 and 35 °C^30^.

Additionally, some studies have employed genomic and transcriptomic approaches to identify ligninolytic enzymes in diverse bacterial strains. In this context, whole genome sequencing (WGS) has become a critical tool for uncovering bacterial ligninolytic genes, such as those encoding DyP-type peroxidases, multicopper oxidases (laccases), and accessory enzymes involved in aromatic compound metabolism^31^. This technique enhances the understanding of microbial degradation mechanisms and supports the development of effective bioremediation strategies^32^. For instance, the genome of *Pseudomonas* Hu109A revealed key lignin-degrading genes, including DyP-type peroxidases, laccase, and O-methyltransferases. It also carries gene clusters for both medium- and short-chain polyhydroxyalkanoates (PHA) synthesis, showing its ability to convert lignin into valuable products and its potential role for industrial applications^33^. In another study,

*Klebsiella* spp. strains isolated from lignocellulose-rich environments exhibited over 20 genes linked to lignin degradation pathways (including catalases, peroxidases, glutathione *S*-transferases and thiol peroxidases), with strain P3TM.1 achieving 98% decolorization of MB within 48 h, indicating robust ligninolytic activity^34^.

Ongoing research aims to discover novel dye-degrading strains, optimize degradation conditions, and create bacterial consortia to increase the efficiency of degradation^35^. Yet few studies link bacterial screening with genomic analysis to correlate genetic potential with actual bioremediation process.

The aim of this study is detection and characterization of soil bacteria for a candidate with potential ligninolytic activity in industrial applications, especially in dye effluent decolorization. The study also aims to apply WGS as a powerful tool to identify lignin-metabolizing enzymes and gain deeper insights into their genetic and functional potential.

## Materials and methods

### Source of bacterial isolates

The bacterial isolates were recovered from 55 soil samples collected from various agricultural sites throughout Egypt including the Rakta Pulp and Paper Company’s effluent treatment facility in Eltalbia, Alexandria, Egypt, these sites are supposed to be rich in ligninolytic bacteria. The soil samples were gathered in sterile plastic bottles of 100 mL volumes. The samples were obtained from the higher layers of the soil, which contain a substantial amount of the microbial community. After collection, samples were kept at 4 °C for subsequent use afterward^36^.

### Isolation of bacteria with potential ligninolytic activities

#### Enrichment and isolates’ recovery

Firstly, the enrichment method was used to allow the bacteria present in low numbers to multiply and reach detectable levels. This was done by the addition of 5 g of soil sample aseptically in a previously autoclaved 250 mL Erlenmeyer flask containing 95 mL minimal salt medium enriched with kraft lignin (KL). The enrichment culture was composed of 1 g/L of commercially available KL (Sigma-Aldrich, USA) and 0.1 g/L yeast extract in phosphate buffered (pH 7) minimal salt medium of the following ingredients (g/L): 4.55 K_2_HPO_4_, 0.53 KH_2_PO_4_, 0.5 MgSO_4_, and 5 NH_4_NO_3_. After soil sample addition, the flasks were placed in an orbital shaking incubator at 30 °C and 200 rpm for one week. Secondly, for isolation of potential ligninolytic bacteria, a series of tenfold serial dilutions were made by adding 1 ml of enriched soil suspension to 9 ml of sterile normal saline and vortexing the mixture followed by transferring 100 μL aliquots of the produced dilutions to surface inoculation of minimal salt medium agar plates containing 1 g/L KL and 0.05 g/L nystatin (to inhibit fungal growth). The plates were then incubated at 30 °C until appearance of colonies, up to one week^37^. The grown colonies were suspected to have potential ligninolytic activity since KL was the sole carbon source in the medium.

#### Purification and storage of bacterial isolates

Based on the variation of colony morphology, individual colonies were picked up and purified using the streak plate technique on minimal salt medium agar plates containing KL. Multiple subcultures were performed to obtain colonies that are pure and distinct^38^. The recovered isolates were kept in 25% glycerol at −80 °C for further investigation.

### Assessment of the recovered isolates for their ligninolytic activities using dye decolorization assays

#### Solid phase dye decolorization assay

This assay was performed by using some indicator dyes to evaluate the ability of the bacterial isolate to decolorize the dye. Luria–Bertani (LB) agar plates containing dye to final concentrations of 25 mg/L for each of Azure B (AB) and methylene blue (MB) or 50 mg/L for congo red (CR) were prepared. All of the indicator dyes were filter sterilized before being aseptically added to the autoclaved media^39^. Pure colonies of each tested isolate were streaked onto the LB agar plates containing dye. Following that, the plates were incubated for five days while being checked each day for the appearance of decolorization zones and/or stained growth (dye adsorption by the growth).

#### Liquid phase dye decolorization assay

The bacterial isolates which had the ability to decolorize all the indicator dyes used in the previous assay, were subjected to the liquid phase dye decolorization assay. For each test isolate, single colonies from the growth obtained on LB plate were used for separate inoculation of 250 mL conical flasks containing 25 mL aliquots of LB broth. The flasks were placed in an orbital shaking incubator at 30 °C and 200 rpm until reaching the exponential phase, then pre-filtered sterilized dyes were aseptically added separately to the flasks at final concentrations of 6 mg/L for each of AB and MB or 40 mg/L for CR. Incubation was resumed for an additional 2 days under the same conditions. Control flasks without bacterial inoculation were included to account for spontaneous dye decolorization. After the completion of the experiment, samples were collected and subjected to centrifugation at 15,000 rpm for 10 min. The absorbance of each sample was estimated spectrophotometrically (Jenway 6800 UV/Vis spectrophotometer, UK) and the produced decolorization of the test dye was calculated as a percentage of its λ_max_ absorbance using the equation shown below (Eq. 1). AB was measured at 650 nm and MB at 665 nm while CR at 470 nm.

$$\mathbf{D}\mathbf{e}\mathbf{c}\mathbf{o}\mathbf{l}\mathbf{o}\mathbf{r}\mathbf{i}\mathbf{z}\mathbf{a}\mathbf{t}\mathbf{i}\mathbf{o}\mathbf{n}(\mathbf{\%})=\frac{{\varvec{A}}{\varvec{b}}{\varvec{s}}{\varvec{o}}{\varvec{r}}{\varvec{b}}{\varvec{a}}{\varvec{n}}{\varvec{c}}{\varvec{e}}\left({\varvec{c}}{\varvec{o}}{\varvec{n}}{\varvec{t}}{\varvec{r}}{\varvec{o}}{\varvec{l}}\right)-\mathbf{A}\mathbf{b}\mathbf{s}\mathbf{o}\mathbf{r}\mathbf{b}\mathbf{a}\mathbf{n}\mathbf{c}\mathbf{e}(\mathbf{t}\mathbf{e}\mathbf{s}\mathbf{t})}{{\varvec{A}}{\varvec{b}}{\varvec{s}}{\varvec{o}}{\varvec{r}}{\varvec{b}}{\varvec{a}}{\varvec{n}}{\varvec{c}}{\varvec{e}}({\varvec{c}}{\varvec{o}}{\varvec{n}}{\varvec{t}}{\varvec{r}}{\varvec{o}}{\varvec{l}})}\times 100$$

Additionally, the visual examination of the pellet’s color after centrifugation was checked to determine whether the dye had been adsorbed to the cells rather than being degraded. The experiment was carried out in duplicate^40^.

#### Bacterial identification by 16S rRNA

This was carried out for the bacterial isolates which exhibited the highest percentage of dye decolorization in liquid phase assay. The purified cultures of the tested isolates were sent to Biotech Serve Co. (Giza, Egypt), for DNA extraction, amplification of the 16S rRNA gene, and sequencing using the Sanger dideoxy sequencing method. The two primers used for PCR amplification of the 16S rRNA gene were universal bacterial primers 27F (5′-AGA GTT TGA TCM TGG CTC AG-3′) and 1492R (5′-TAC GGY TAC CTT GTT ACG ACT T-3′). The NCBI BLAST (BASIC LOCAL ALIGNMENT SEARCH TOOL; http://blast.ncbi.nlm.nih.gov/Blast.cgi) was used to assess DNA similarity and sequence alignment. The sequences with the highest identity score were then retrieved and aligned using multiple sequence alignment tool with the CLUSTAL W of MEGA software (Version 11.0.13). Then, the aligned sequences were used for the preparation of a phylogenetic tree using the same software (MEGA) following the maximum likelihood method^41^.

#### Estimation of extracellular and intracellular ligninolytic activity for the test isolate

The dye was incubated with either the culture supernatant or crude cell lysate of the isolate that exhibited the highest activity in the previous assay to assess extracellular and intracellular activity, respectively. The experiment was carried out by culturing the isolate in both LB broth and lignin containing medium to induce the lignin degrading/dye-decolorizing enzymes^40^. Two pure colonies of the bacterial isolate were used for separate inoculation of 250 mL flasks containing 25 mL of either LB broth or Mineral Salt Medium supplied with 600 mg/L KL (MSM-KL), 10 g/L glucose, and 3 g/L peptone. The MSM was composed of the following components in g/L: K_2_HPO_4_, 2.0; Na_2_HPO_4_, 2.4; MgSO_4_.7H_2_O, 0.01; CaCl_2_, 0.01 and a trace element solution of 1.0 mL/L^36^. After that, the flasks were placed in an orbital shaking incubator at 30 °C and 200 rpm for 3–4 days. At the end of the incubation period, the contents of each flask were centrifuged at 4 °C and 7000 rpm in a cooling centrifuge for separation of culture biomass from culture supernatant. The biomass was subjected to sonication for the preparation of crude cell lysate. Both cell-free culture supernatant and crude cell lysate were used for determination of extracellular and intracellular ligninolytic activities, respectively as shown below.

#### Testing extracellular decolorizing activity of the test isolate

The decolorizing activity was carried out using the cell-free culture supernatant^42^. The reaction mixture was composed of 0.5 mL of the culture supernatant, 0.5 mL of 0.160 mM AB, and 1.5 mL of 125 mM sodium tartrate buffer (pH 3.0) and the assay was carried out in test tubes. To start the reaction, 0.5 mL of 2 mM hydrogen peroxide was added to each test tube^43^. Static and shaking conditions were both carried out for the reaction over a period of 2 days. Control flasks were included to account for spontaneous dye decolorization. The absorbance of each sample was estimated spectrophotometrically at λ_max_ (650 nm) and the produced decolorization activity was calculated as a percentage relative to control using Eq. (1).

#### Testing the intracellular decolorizing activity of the test isolate using the crude cell lysate

After harvesting the cell biomass from each flask, the cells were washed using phosphate buffered saline (PBS) and resuspended in 5 mL of PBS, sonicated by using an ultrasonic processor with an amplitude of 50% for 5–10 min, in cycles of 30 s on and 30 s off, while maintaining the sample on ice to prevent overheating. After that, the sample was centrifuged at 15,000 rpm for 5 min. The supernatant was collected, recentrifuged under the same condition and this process was repeated several times to remove any cell debris. The obtained clear supernatant representing the crude cell lysate was used for measuring the decolorization activity. The assay was carried out in test tubes and the reaction mixtures were composed of certain volumes of crude cell lysate (0.1, 0.5, 1, and 2 mL). To each reaction mixture, 0.5 mL of 0.160 mM AB was added, and the volume was completed with PBS to 2.5 mL. To start the reaction, 0.5 mL of 2 mM hydrogen peroxide was added to each reaction mixture. Static and shaking conditions were both carried out for the reaction over a period of 2 days. Control flasks were included to account for spontaneous dye decolorization. The assay was completed as mentioned earlier.

#### Estimation of decolorizing activity of the test isolate under growth conditions

Single colonies were used for separate inoculation of 250 mL conical flasks containing 25 mL aliquots of either LB broth or MSM-KL supplied with 10 g/L of glucose, and 3 g/L of peptone^36^. The flasks were placed in a shaking incubator for 3 days, and then pre-filtered sterilized AB dye was aseptically added to the flasks at final concentrations of 6 mg/L. Incubation was resumed for an additional one day. Control flasks without bacterial inoculation were included to account for spontaneous dye decolorization^40^. Samples were withdrawn at various time intervals (0, 4, and 24 h) following dye addition and subjected to centrifugation at 15,000 rpm for 10 min. The absorbance of each sample was estimated spectrophotometrically at λ_max_ (650 nm) and the produced decolorization activity was calculated as a percentage relative to control using Eq. (1).

### Effect of different factors on growth and decolorizing activity of the test isolate using one-factor-at-a-time approach (OFAT)

#### Preparation of seed and dye biodegradation flasks

Fresh seed culture (1–3 × 10^6^ CFU/mL) was prepared for each parameter under investigation and used to inoculate dye biodegradation flasks containing 25 mL MSM-KL per 250 mL flask. In each experiment, after the predetermined incubation period, prefiltered AB dye was added at 6 mg/L and the flasks were re-incubated under the same growth conditions for 1 additional day. Samples were withdrawn at different time intervals (0, 4, and 24 h) following AB addition for determination of dye decolorization activity and microbial growth by dry cell weight (DCW) method. The decolorizing activity was performed by the quantitative assay and expressed as a percentage following the previously mentioned Eq. (1). The uninoculated medium was used as a control in all cases. All experiments were done in duplicate.

#### Bacterial growth estimation by DCW

To determine the DCW, 1.5 mL aliquots were collected in pre-weighed centrifuge tubes and centrifuged, then the supernatants were carefully aspirated out while the cell pellets were dried at 70 °C till constant weight. After that, each tube was weighed to determine the dry weight of the cells by subtracting the initial weight from the final weight of each centrifuge tube^44,45^. The DCW was calculated and expressed as g/L.

#### Studying the effect of nutritional and environmental factors

In the conventional scaling-up approach, various nutritional and physical parameters were optimized by maintaining all factors at a constant level in the basal medium, except the one under study. Each subsequent factor was examined after taking into account the previously optimized factor(s)^46^. The impact of several parameters on the decolorizing activity of the selected isolate was investigated. The factors and their ranges were tested as follows:

**Inoculum size (% v/v):** 1, 1.5, 2, and 2.5.

**Incubation time (day)**: 1, 2, 3, 4, 5, and 6.

**Agitation speed (RPM):** 120, 160 and 200.

**Temperature (ºC):** 25, 30, and 35.

**Carbon source (0.4% w/v):** glucose, sucrose, starch, and the effect of no addition of extra carbon source was also assessed.

**Lignin concentration (mg/L):** 200, 400, 600, 800, and 1000.

**Nitrogen source (0.4% w/v):** tryptone, urea, peptone, ammonium nitrate, and yeast extract.

**Initial pH of the fermentation medium:** 6, 8, 10, and 12.

#### Optimization of dye decolorizing activity using design expert software

Response surface methodology (RSM), central composite design (CCD) was applied to optimize the environmental factors for the dye decolorization assay. Because the OFAT approach to optimization is time-consuming and tedious, RSM with CCD was selected for appropriate variation of key components. The model for decolorization of AB was implemented with the identification of likely interactions and optimal operating parameters. The effects of four independent factors, including lignin concentration (A), tryptone concentration (B), pH (C), and incubation time (D), each with three levels (low, medium, and high), on one response % decolorization of the dye as a dependent variable were examined in a total of 30 experiments. To determine the percentage of dye decolorization after four hours of dye addition, the combined impacts of four factors were examined in the designated ranges including lignin concentration (400–600 mg/L), tryptone concentration (1–7 g/L), pH (8–10), and incubation period (24–72 h).

Table 1 also displays the experimental design matrix produced from the CCD model using DESIGN EXPERT (DESIGN EXPERT Software, v. 13.0.5.0, Stat-Ease Inc., Statistics Made Easy, Minneapolis, MN, USA) which contributed to the final model equation with observed and predicted responses.

**Table 1:** The central composite design (CCD) runs in design expert Software for three selected levels of the four tested factors including lignin conc, tryptone conc, initial pH, and incubation time.

Run order	A: Lignin conc (mg/L)	B: Tryptone conc (g/L)	C: pH	D: Incubation time* (h)	Decolorization of dye (%)	
					Observed response	Predicted response
1	400	7	10	72	16.213	22.89
2	400	7	8	72	33.6	27.38
3	500	4	9	48	43.811	57.95
4	500	4	9	48	55.263	57.95
5	400	7	10	24	48.79	48.16
6	500	4	9	48	58.036	57.95
7	400	1	8	72	4.44	3.01
8	500	4	11	48	50.976	59.29
9	500	4	9	0	2.394	8.30
10	600	7	8	24	49.86	50.11
11	500	4	7	48	57.578	56.61
12	400	1	10	72	0.209	-1.49
13	600	1	8	24	46.82	42.94
14	500	4	9	48	57.31	57.95
15	600	7	10	72	37.168	32.02
16	500	4	9	48	62.518	57.95
17	600	7	10	24	61.015	57.28
19	500	-2	9	48	4.936	15.59
20	500	10	9	48	51.892	47.15
21	600	1	10	24	53.301	50.11
22	300	4	9	48	52.227	50.25
24	400	7	8	24	38.029	40.98
25	500	4	9	48	64.332	57.95
26	600	1	10	72	12.217	4.79
27	400	1	10	24	49.788	43.84
28	600	1	8	72	9.376	9.28
29	600	7	8	72	27.082	36.51
30	400	1	8	24	40.201	36.66

*Growth age before dye addition.

The dye biodegradation media (MSM) of the RSM model-based experiments were prepared and distributed as 25 mL aliquots in 250 mL Erlenmeyer flasks. They were inoculated at 1.5% v/v mL of the seed culture and then incubated at the previously found optimum agitation speed and temperature. After incubation, samples were collected following four hours of dye addition to be tested for dye decolorization activity of the test isolate.

#### Statistical and graphical investigations

To determine how well the model fits the data, analysis of variance (ANOVA) was examined which included estimation of *p*-values, lack of fit F-values, adjusted and predicted R^2^. The model reduction was also implied to improve model statistics. Only factors with *p*-values lower than 0.05 were considered significant. To ensure model fitness, lack of fit *p*-value must be greater than 0.1. To illustrate the distinct and combined effects of parameters on the response, 3D response surface and contour graphs were displayed.

#### Validation of the applied model data

The final model’s equation and an examination of RSM plots were used to figure out and predict the maximum response values (highest decolorization percentages). Two optimized confirmation experiments were run, and the findings were compared with the values predicted by the equation to assess the model’s accuracy. The results obtained under optimum conditions and those obtained under non-optimized conditions were also compared.

### Detection of genes responsible for ligninolytic activity

#### Genomic DNA extraction

DNA extraction was carried out using Quick-DNA™ Bacterial Miniprep kit according to the manufacturer’s instructions.

#### Quality control pre-tests

The genomic extract of the test isolate was then sent to Beijing Genomics Institute (BGI) Hongkong Tech Solution NGS Lab (Tai Po, New Territories, Hong Kong) by DHL to perform the whole genome sequencing process (WGS). Upon arrival, some quality control pretests were done such as determination of sample concentration, purity, and integrity. Concentration was detected by a fluorometer (e.g., Qubit Fluorometer, Invitrogen). Sample integrity and purity were detected by agarose gel electrophoresis (concentration of agarose gel: 1% voltage: 150 V, electrophoresis time: 40 min).

#### Genome sequencing and assembly

The genomic DNA extract of the selected test isolate was sequenced using a DNBSEQ G400 PE150 (MGI Tech Co., Ltd). The raw reads of low quality from paired-end sequencing (those with consecutive bases covered by fewer than five reads) were discarded. The sequenced reads were then assembled using Spades v 3.15.2 software.

#### Bioinformatic analysis for detection of genes responsible for ligninolytic activity

The bioinformatics analysis was proceeded for annotation and detection of genes responsible for ligninolytic activity. Genome annotation is the process of identifying functional elements along the sequence of a genome. The FASTA file of clean reads after assembly was uploaded to BACTERIAL AND VIRAL BIOINFORMATICS RESOURCE CENTER BV-BRC (https://www.bv-brc.org/) for annotation of genes. The annotation service available in BV-BRC uses a modular, updated version of rapid annotation using subsystem technology (RAST)^47^ that is called the RAST toolkit (RASTtk)^48^. Genes related to lignin degradation and dye decolorization activity were identified and manually annotated by performing a BLASTP search against the ‘nr’ database.

#### Submission of the next generation sequencing (NGS) reads to the national center for biotechnology information (NCBI)

The FASTQ files of Next Generation Sequencing (NGS) readings were submitted to the National Center for Biotechnology Information (NCBI) to SRA database (https://submit.ncbi.nlm.nih.gov/about/sra/).

## Results

### Isolation of bacterial isolates with potential ligninolytic activity

A total of 177 possible ligninolytic bacteria were retrieved from 55 soil samples that were collected from different locations in Egypt. Coded numbers were assigned to soil samples and bacterial isolates (Table 2).

**Table 2:** Isolates recovered from the collected soil samples and their assessment for ligninolytic activity using solid phase dye decolorization assay.

Soil code	Isolate code	Decolorization appeared on LB-dye agar plates containing:		
		AB*	MB*	CR*
A1	11	+(a)	−	+(a)
	13	+(a)	−	−
	14	−	−	−
	15	−	+(a)	−
	16	−	−	−
A2	23	+(a)	−	−
	24	−	−	−
	25	−	−	−
	26	+(a)	+(a)	−
	27	+(a)	+(a)	+(a)
A3	31	−	+(a)	−
	32	−	−	−
	33	−	−	+(a)
A4	41	−	−	−
	42	+(a)	−	+(a)
	43	+(a)	+(a)	+(a)
	44	+(a)	−	−
	45	+	+	+(a)
A5	51	+(a)	+(a)	−
	52	+(a)	−	−
	53	−	+(a)	+(a)
	54	−	+(a)	+(a)
	55	+(a)	+(a)	+(a)
M6	61	−	+(a)	−
	62	−	−	−
	63	−	−	−
	64	−	−	−
	65	−	−	+(a)
	66	−	+(a)	−
	67	+(a)	−	+(a)
M7	71	−	−	+(a)
	72	−	−	−
	73	+(a)	−	+(a)
	74	+(a)	+ (a)	+(a)
	75	−	−	−
	76	+(a)	−	−
M8	81	−	−	−
	82	−	−	+(a)
	83	−	−	−
C9	92	−	−	−
	93	−	−	−
C10	101	−	−	−
	102	−	+(a)	+(a)
C11	111	−	+(a)	+(a)
	112	−	−	+(a)
C12	121	−	−	−
C13	131	−	+(a)	−
	132	−	−	−
	133	−	−	−
	134	−	−	−
C14	141	−	+(a)	−
	142	−	−	−
G15	151	−	−	−
	153	−	−	−
	154	+(a)	+(a)	−
G16	161	−	−	+(a)
	163	−	−	+(a)
	164	+	+	−
U17	171	−	−	+(a)
	173	−	−	−
U18	182	−	−	−
	183	−	−	−
B19	191	−	−	−
	192	+(a)	+(a)	−
B20	202	−	−	−
	203	−	−	−
B21	214	+(a)	−	−
B22	223	−	−	−
	224	−	+(a)	−
B23	231	−	−	+(a)
	232	−	−	−
G24	242	−	−	−
G25	252	−	−	−
	253	+(a)	+(a)	+(a)
	254	−	+(a)	−
G26	261	−	−	−
	262	−	−	+(a)
	263	−	−	+(a)
	264	−	−	−
B27	272	−	−	−
	273	−	−	−
F28	281	+(a)	+(a)	−
	282	−	−	−
F29	291	−	−	−
	292	−	−	−
F30	301	−	+(a)	−
	302	−	−	−
	303	−	−	−
	304	+	+	+(a)
	305	−	−	−
F31	311	−	+(a)	−
	312	−	+(a)	−
	313	+(a)	+(a)	−
	314	−	+(a)	−
U32	321	+(a)	+(a)	−
	323	−	−	+(a)
	324	−	−	−
U33	331	+(a)	−	−
	332	−	−	−
	333	+	+	+(a)
	334	−	+(a)	−
	335	−	−	−
	336	−	−	−
U34	342	−	−	−
	344	−	−	−
H35	351	+(a)	+(a)	−
	352	−	−	−
P36	361	+(a)	−	+(a)
	362	+(a)	−	−
	364	−	−	−
	365	−	−	−
P37	372	+(a)	−	+(a)
	373	−	+(a)	−
	374	−	−	−
	375	−	+(a)	−
	376	−	−	−
P38	381	−	−	−
	382	−	+(a)	−
	383	−	−	−
	384	−	−	−
	389	−	−	−
P39	391	+	+	+(a)
	392	+(a)	+(a)	−
	393	+(a)	+(a)	−
G40	401	−	−	−
H41	411	−	+(a)	+(a)
	412	−	+ (a)	−
	413	−	−	−
	414	−	−	+(a)
	415	−	−	−
	416	+(a)	+(a)	+(a)
H42	421	+(a)	+(a)	−
	422	−	+(a)	+(a)
H43	431	−	−	−
	432	−	−	−
	433	−	+(a)	−
	434	+	+	+(a)
H44	441	−	−	−
	442	−	−	−
	443	−	−	+(a)
	444	−	−	+(a)
	445	−	−	−
	446	−	−	+(a)
H45	451	−	+(a)	−
	452	−	−	+(a)
	453	+	−	+(a)
	454	−	−	−
	455	−	−	−
	456	−	−	+(a)
	457	−	+(a)	+(a)
H46	461	−	−	−
	462	−	+(a)	−
	463	−	−	−
H47	471	−	+(a)	−
	472	+(a)	+(a)	−
	473	−	−	+(a)
	474	−	−	−
G48	481	−	−	−
Q49	492	−	−	−
	493	−	−	+(a)
	495	+	+	+(a)
Q50	501	+(a)	+(a)	+(a)
Q51	511	+	+	+(a)
Q52	521	−	+(a)	−
	522	+	+	+(a)
	523	−	−	−
	524	−	−	+(a)
Q53	531	−	−	−
	534	−	−	−
	535	−	−	−
Q54	541	−	+(a)	−
	542	+(a)	−	−
	543	−	+ (a)	−
	544	+(a)	+ (a)	+(a)
Q55	551	−	−	−
	552	+(a)	−	−
	553	−	−	−

+ : positive; − : negative; MB: methylene blue; AB: Azure B; CR: Congo red; a: isolates showed adsorption of dye on growth surface.

(A): Alexandria; (B): Banha; (C): Cairo; (F): Faiyum; (G): Giza; (H): Helwan; (M): Menoufia; (P): Port Said; (Q): Qalyubia; (U): Upper Egypt.

### Assessment of the recovered isolates for their ligninolytic activities using dye decolorization assays

#### Solid phase dye decolorization assay

**Fig. 1:**
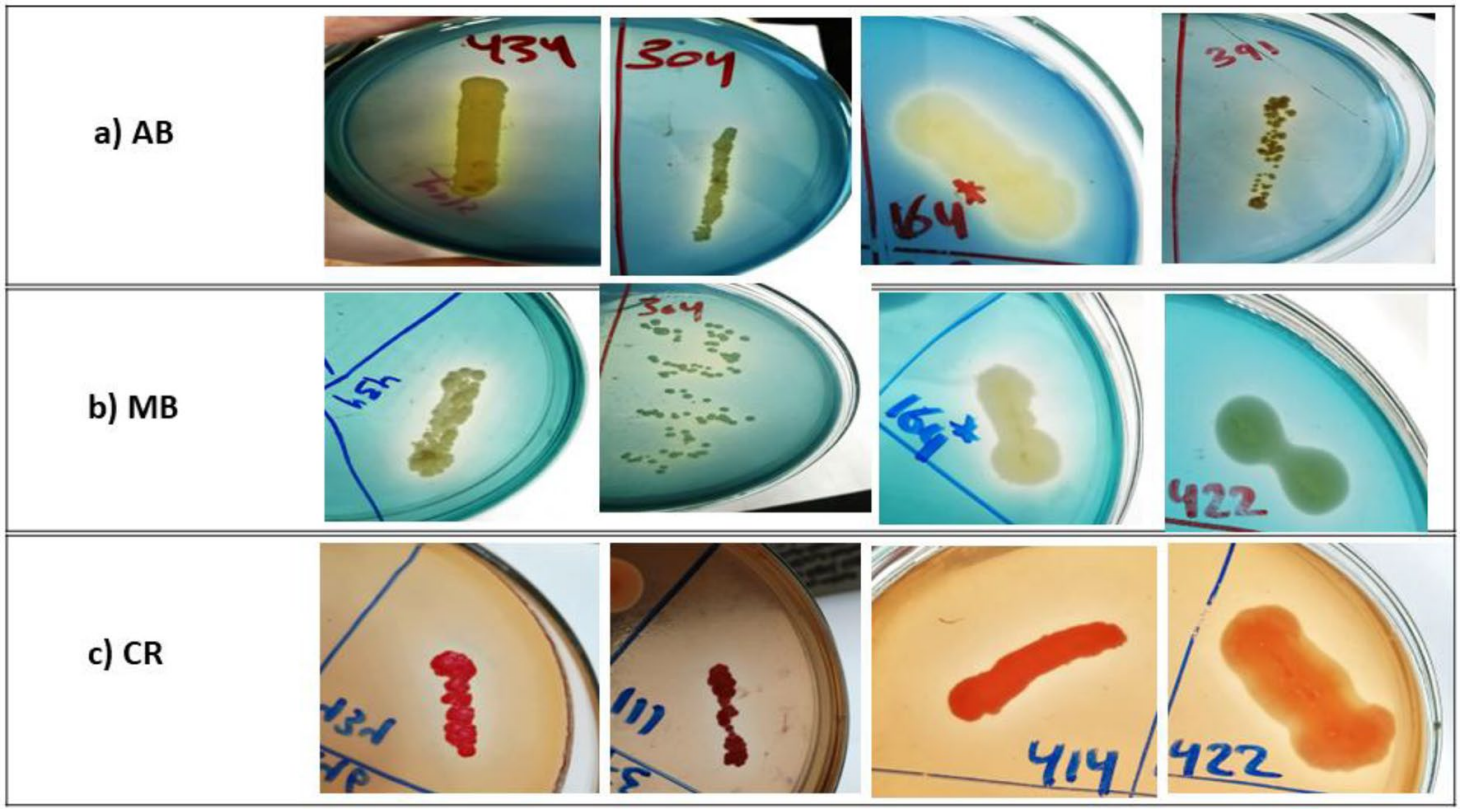
Decolorization zones of some representative isolates with potential ligninolytic activity on LB-dye agar plates. (**a**) AB (Azure B) for isolates 434, 304, 164, and 391; (**b**) MB (methylene blue) for isolates 434,304, 164, and 422; (**c**) CR (Congo red) for isolates 434, 111, 414, and 422.

Dye decolorization assay in solid agar plates revealed that 46 (25.9%), 60 (33.8%), and 51 (28.8%) of bacterial isolates showed decolorization zones with AB, MB, and CR, respectively (Fig. 1 and Table 2). As demonstrated in Table 2, some isolates on AB and MB plates showed dye adsorption, while others did not. However, all the tested isolates in the CR assay had adsorbed the CR dye on the growth surface.

#### Liquid phase dye decolorization assay

**Fig. 2:**
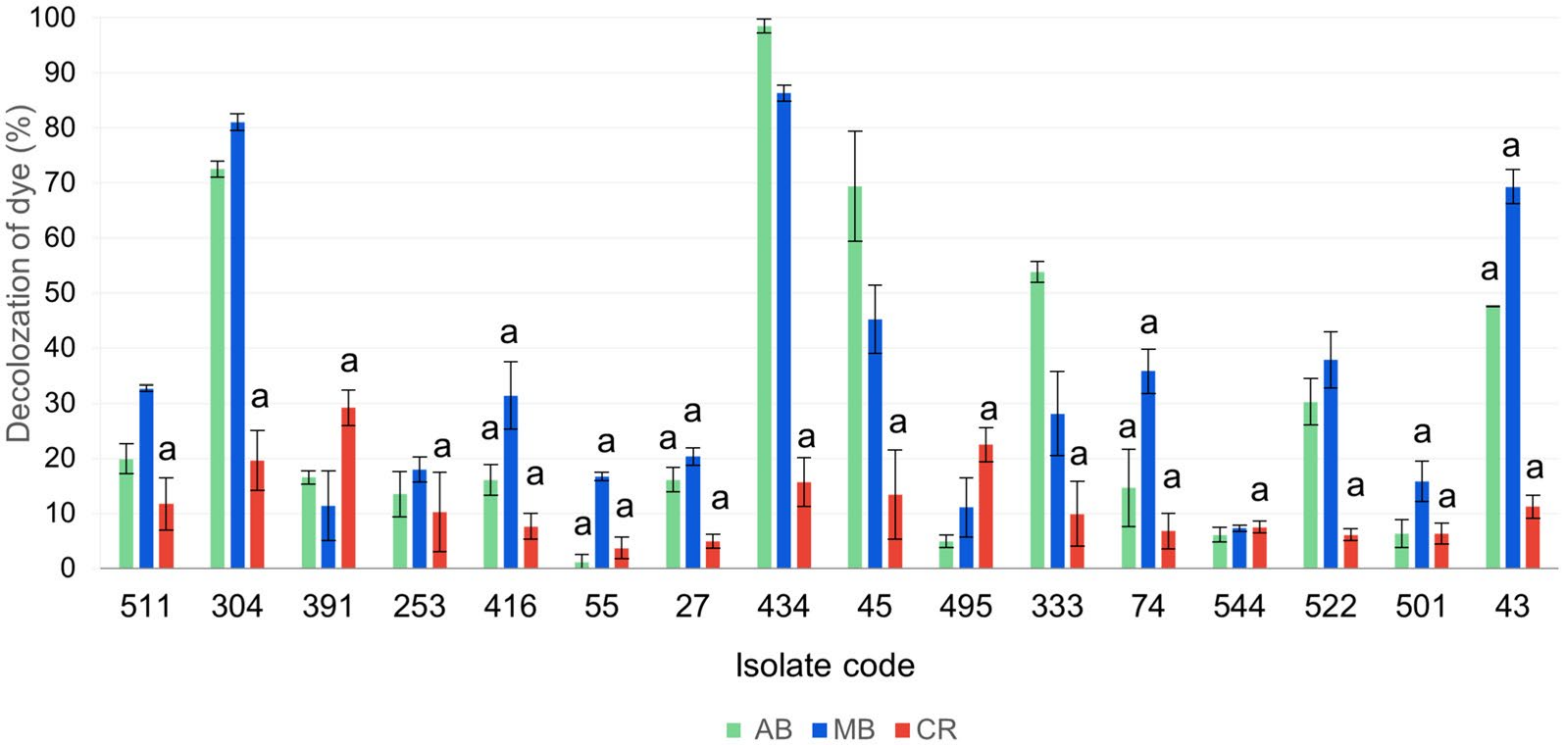
Decolorization percentages produced by the tested isolates in LB broth containing Azure B (AB), methylene blue (MB), or Congo red (CR) as determined in liquid phase assay. (**a**) Isolates showed adsorption of dye on pellets’ surface after centrifugation.

Liquid phase assays were carried out for a total of 16 bacterial isolates which gave positive results on the three different types of dyes in solid phase assay (Table 3). The liquid assay of AB showed that isolates 434 and 304 have the highest decolorization capacity with percentages of 98.5 and 72.5%, respectively, and of 86.3 and 81%, respectively in MB assay after 2 days of incubation with the dye. However, in the CR assay, isolate 391 showed the highest decolorization activity by 29.2% (Fig. 2). Dye adsorption on the pellets’ surface after centrifugation was also monitored and the results are shown in (Fig. 2). Similar to the results obtained in solid phase dye decolorization assay, all the tested isolates in CR assay showed adsorbed dye on the pellets’ surface.

**Table 3:** Summarized results showing bacterial isolates with decolorization capacity on all methylene blue-, Azure B- and Congo red- LB agar plates.

Soil code	Isolate code	Decolorization capacity for:		
		AB*	MB*	CR*
A2	27	+(a)	+(a)	+(a)
A4	43	+(a)	+(a)	+(a)
	45	+	+	+(a)
A5	55	+(a)	+(a)	+(a)
M7	74	+(a)	+(a)	+(a)
G25	253	+(a)	+(a)	+(a)
F30	304	+	+	+(a)
U33	333	+	+	+(a)
P39	391	+	+	+(a)
H41	416	+(a)	+ (a)	+(a)
H43	434	+	+	+(a)
Q49	495	+	+	+(a)
Q50	501	+(a)	+ (a)	+(a)
Q51	511	+	+	+(a)
Q52	522	+	+	+(a)
Q54	544	+(a)	+ (a)	+(a)

+ : positive; − : negative; MB*: methylene blue; AB*: Azure B; CR*: Congo red; a: isolates showed adsorption of dye on their growth surfaces.

(A): Alexandria; (F): Faiyum; (G): Giza; (H): Helwan; (M): Menoufia; (P): Port Said; (Q): Qalyubia; (U): Upper Egypt.

#### Molecular identification of bacterial isolates

**Fig. 3:**
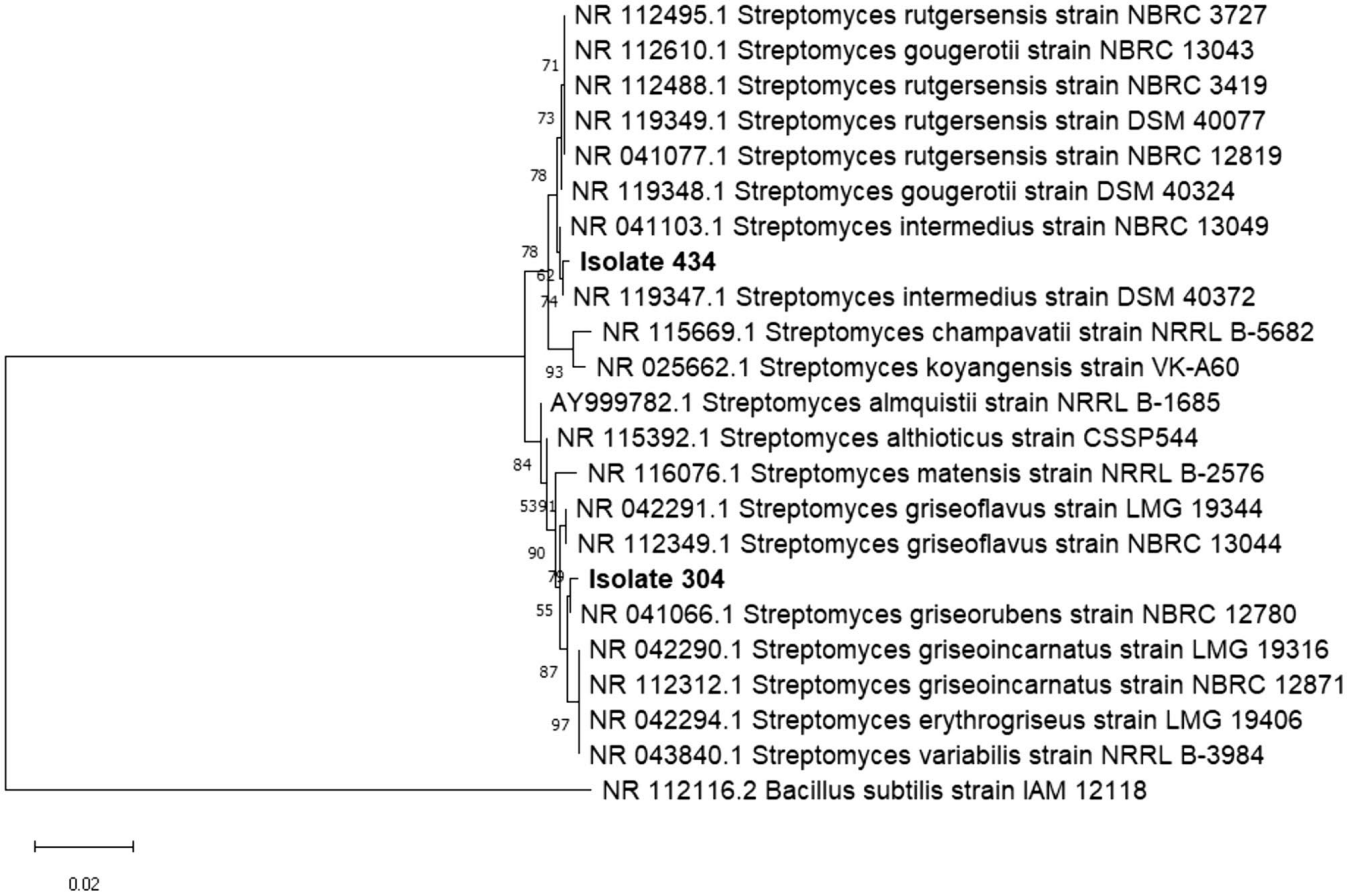
The phylogenetic tree of isolates 434, and 304 based on 16S rRNA genes sequence.

The 16S rRNA gene was sequenced to identify two selected isolates (434 and 304) that had the highest decolorization activity for the tested dyes. Isolate 434 could be identified as *Streptomyces intermedius* since it showed 99.86% homology of the 16S rRNA nucleotide sequences with *Streptomyces intermedius* strain DSM 40372 (NR_119347.1). While isolate 304 could be identified as *Streptomyces griseorubens* with 99.93% homology of the 16S rRNA nucleotide sequence of *Streptomyces griseorubens* strain NBRC 12780 (NR_041066.1). The partial nucleotide sequences of 16S rRNA genes of the two isolates (434 and 304) were submitted and deposited in the NCBI GenBank database under the accession numbers OQ928560 and OQ928561, respectively. According to the phylogenetic tree (Fig. 3), *S. intermedius* and *S. griseorubens* are the closest strains in similarity to the tested isolates, 434 and 304, respectively.

### Extracellular and intracellular ligninolytic activity for the test isolate

#### Extracellular decolorizing activity of the test isolate

None of the culture supernatants from either LB or lignin containing medium grown culture exhibited significant dye-decolorizing ability (not more than 10% decolorization could be detected) for either static or shaking assay conditions after 2 days of incubation with the dye.

#### Intracellular decolorizing activity of the test isolate using the crude cell lysate

The results for the crude cell lysate obtained from LB culture showed no decolorizing activities after 2 days of incubation with the dye, while the corresponding results of lignin containing medium’s culture showed slightly decolorizing activities after 2 days of incubation with the dye (less than 5% decolorization).

#### Decolorizing activity of the test isolate under growth conditions

After 72 h growth age of *S. intermedius* isolate, the addition of AB dye resulted in decolorization capacity in LB medium of 0.002, 0.06, and 30% and in MSM-KL of 6.8, 21, and 41% following dye addition and re-incubation under the same growth conditions for 0, 4, and 24 h, respectively.

### Effect of different factors on growth and decolorizing activity of the test isolate using one-factor-at-a-time approach (OFAT)

#### Effect of different inoculum sizes

**Fig. 4:**
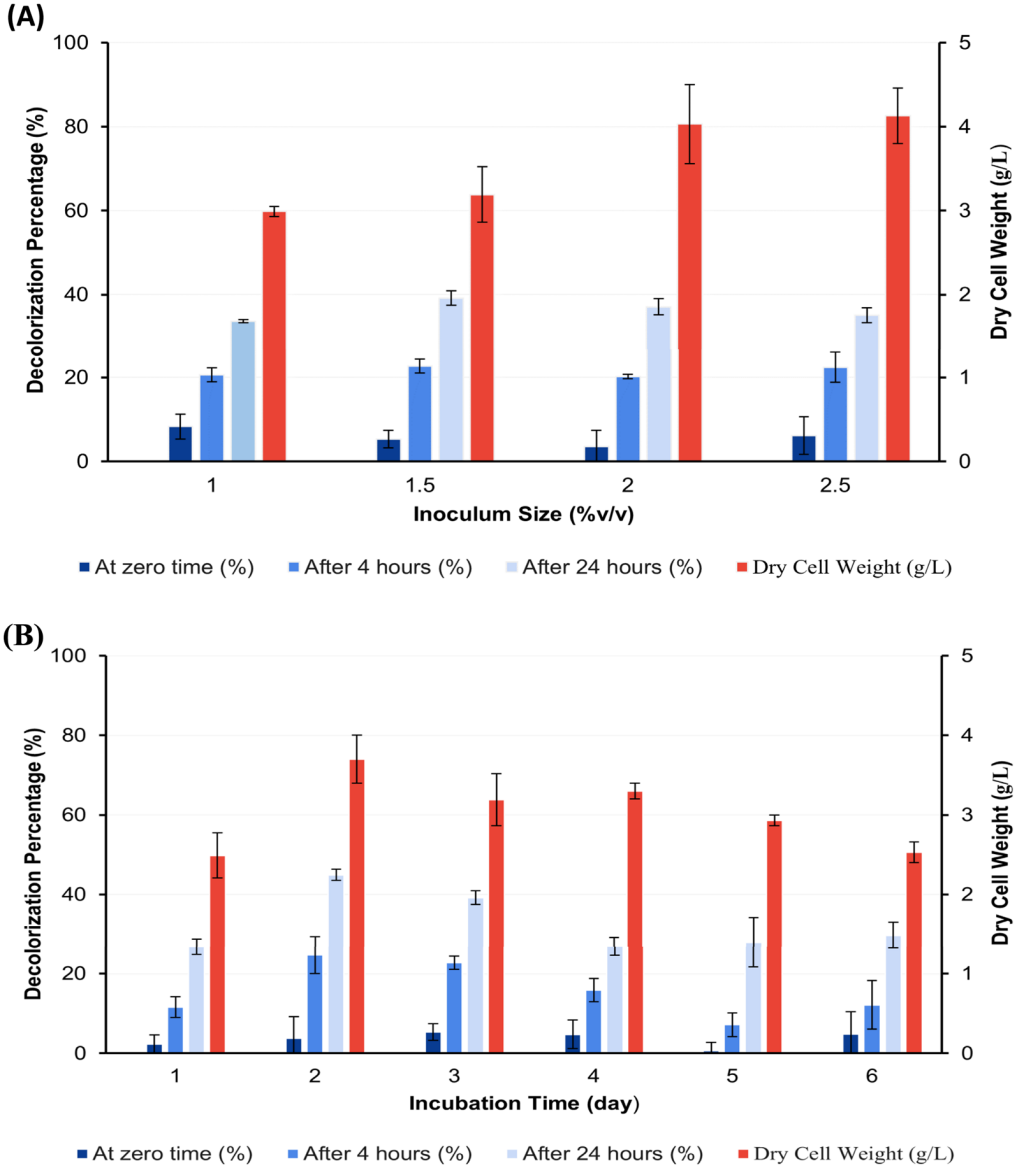
Effect of different inoculum sizes (**A**) and different incubation times (**B**) on decolorization percentage of AB after 0, 4, and 24 h of dye addition to the grown culture of *S. intermedius* test isolate. Lines above columns indicate standard deviation values.

The result of the decolorization assay at 4 and 24 h following the addition of dye to 72 h age culture and re-incubation under the same growth conditions showed that the highest decolorization capacity was 22.69 and 39.18% upon using 1.5% v/v inoculum size, respectively (Fig. 4A).

#### Effect of incubation time

Two days of incubation growth period produced the maximum decolorization activity, which was 24.7 and 44.92% after 4 and 24 h following the addition of dye and re-incubation under the same growth conditions, respectively (Fig. 4B).

#### Effects of agitation speed and incubation temperature

**Fig. 5:**
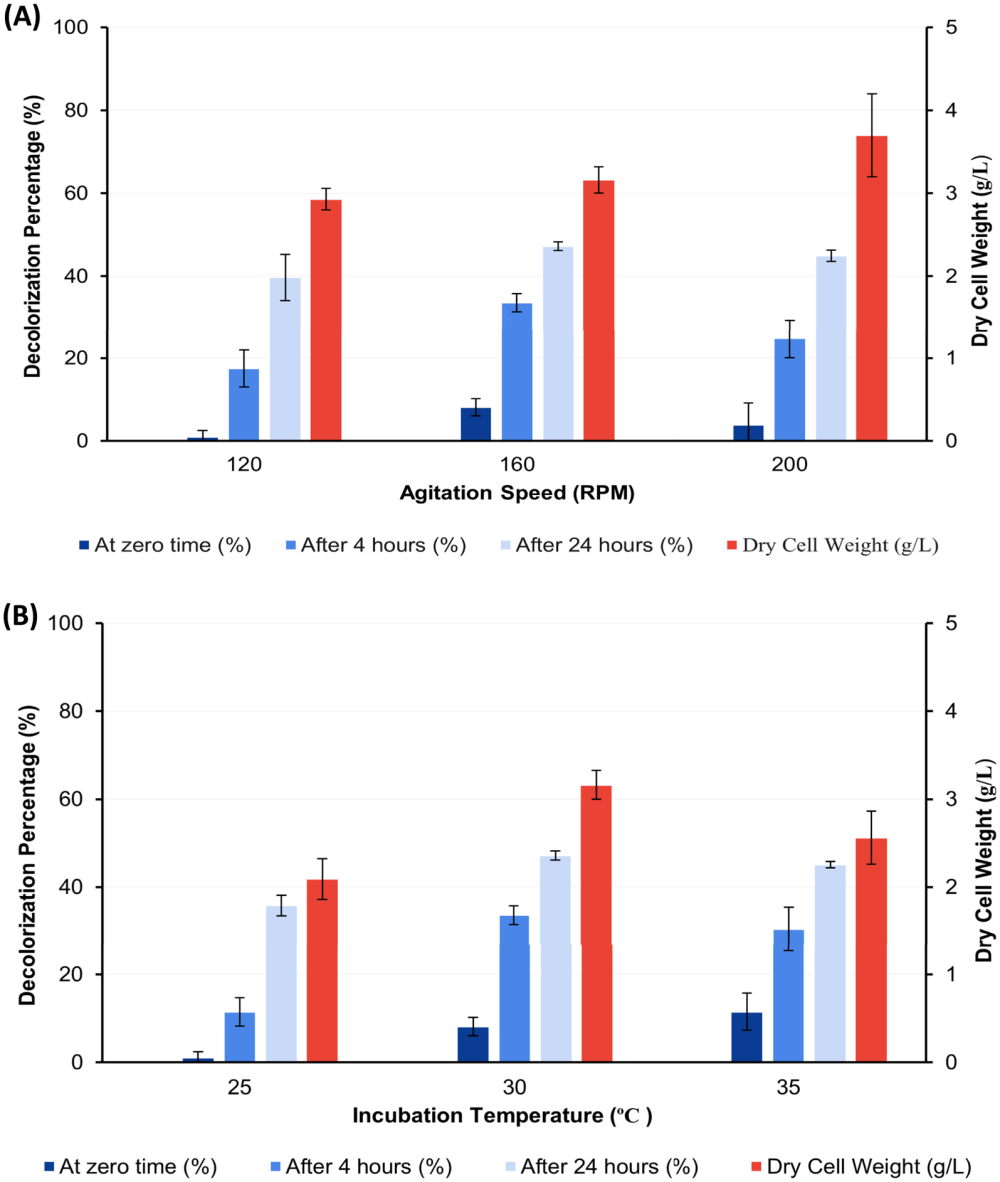
Effect of agitation speed (**A**) and different temperatures (**B**) on decolorization percentage of AB after 0, 4, and 24 h of dye addition to the grown culture of *S. intermedius* test isolate. Lines above columns indicate standard deviation values.

As demonstrated in Fig. 5A,B, the maximum decolorization activity was obtained at 160 rpm and 30 ºC which was 33.58 and 47.18% at 4 and 24 h following dye addition to 2 days age culture and re-incubation under the same conditions, respectively.

#### Effect of carbon source

**Fig. 6:**
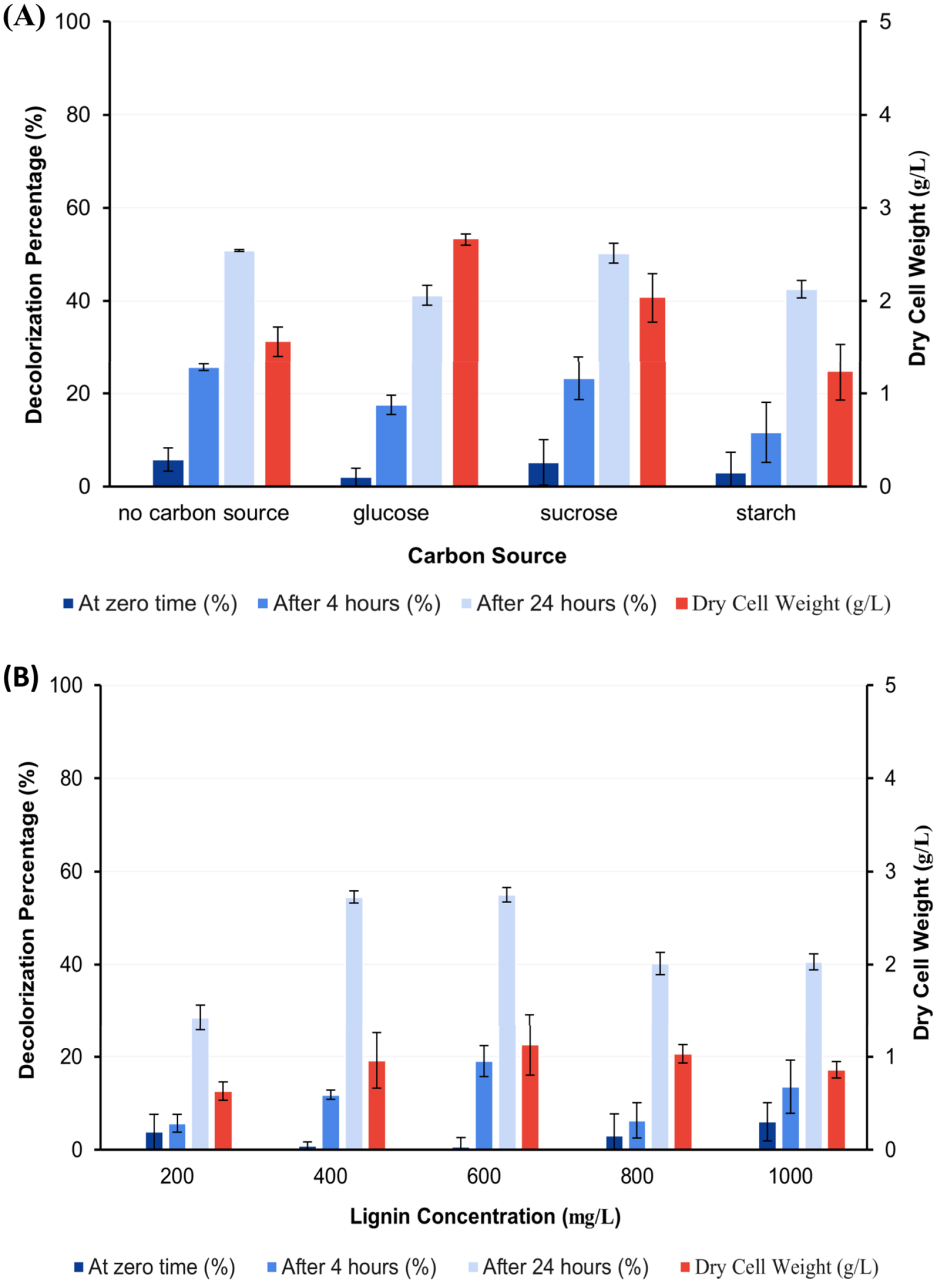
Effect of carbon source (**A**) and lignin concentrations (**B**) on decolorization percentage of AB after 0, 4, and 24 h of dye addition to the grown culture of *S. intermedius* test isolate. Lines above columns indicate standard deviation values.

The maximum activity for dye decolorization was observed by no addition of extra carbon source to MSM-KL. These maximum activities were 25.65 and 50.84% at 4 and 24 h following the addition of dye to 2 days age culture and re-incubation under the same growth conditions, respectively (Fig. 6A).

#### Effect of lignin concentration

The ability to decolorize AB by 19.07 and 54.92% at 4 and 24 h following dye addition, respectively, was demonstrated by a lignin concentration of 600 mg/L (Fig. 6B).

#### Effect of nitrogen source

**Fig. 7:**
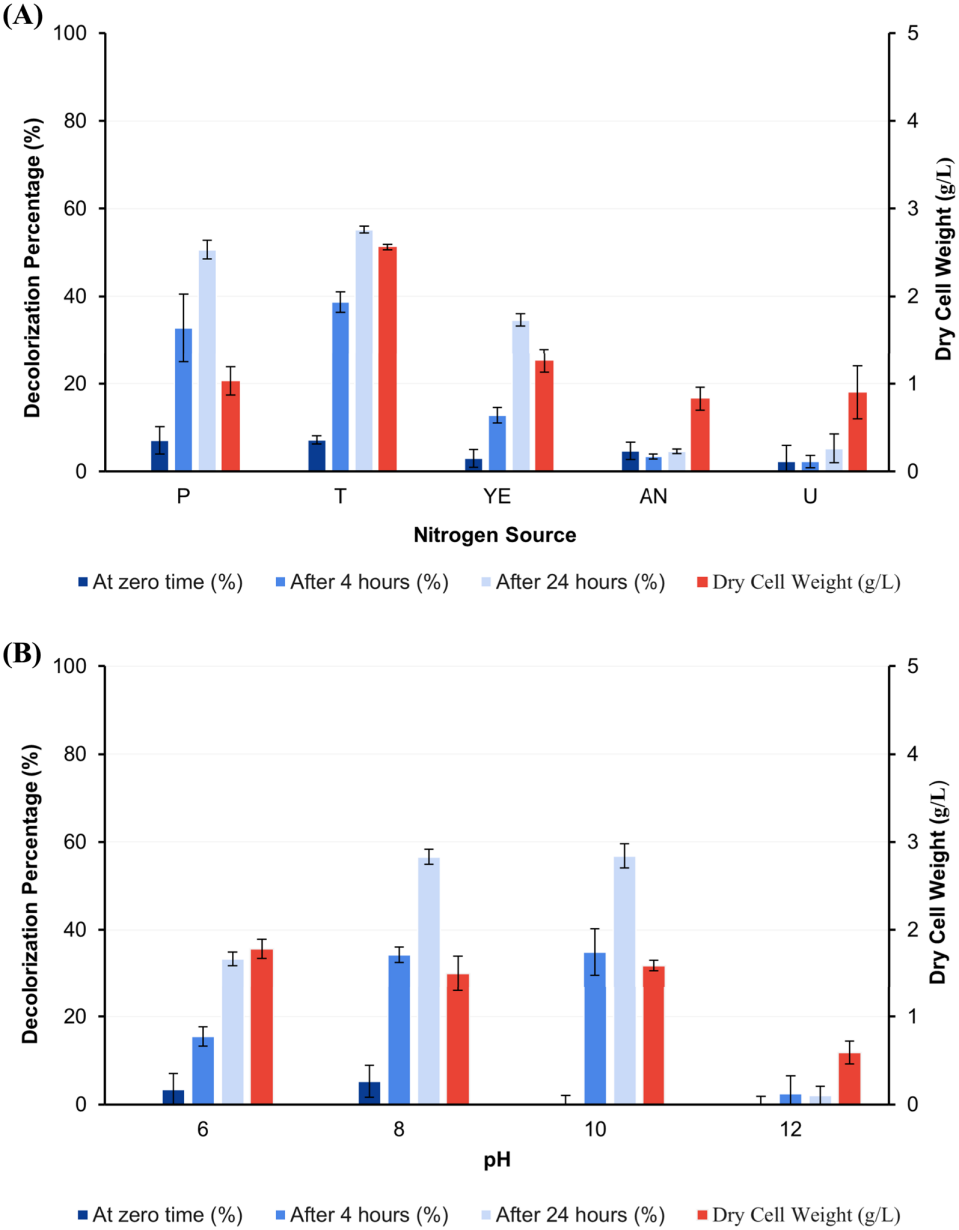
Effect of some nitrogen sources (P: Peptone, T: Tryptone, YE: Yeast extract, AN: Ammonium nitrate, and U: Urea) (**A**) and different pH (**B**) on decolorization percentage of AB after 0, 4, and 24 h of dye addition to the grown culture of *S. intermedius* test isolate. Lines above columns indicate standard deviation values.

The highest activity was obtained when using tryptone as a nitrogen source by 38.69, and 55.17% followed by peptone and yeast extract as shown in Fig. 7A at predetermined assay time measurements.

#### Effect of initial pH

After 4 and 24 h of dye addition, pH 8 and 10 showed close decolorization activity by 34.27, 56.59, and by 34.92, 56.76%, respectively (Fig. 7B).

#### Statistical optimization of process parameters, analysis, and validation

RSM (CCD) was used to perform comprehensive research on dye decolorization by the selected *S. intermedius* test isolate. The design included 16 factorial points, 8 axial points, and 6 repetitions at the center point for the estimation of the pure error sum of squares. Analysis of variance (ANOVA) for percentage decolorization given in Table 4 revealed that the quadratic model is highly statistically significant, as indicated by the model F value of 24.64 which means that there is only a 0.01% chance that the value is significant by chance. the *p-* value is a measure of a test’s statistical significance; a value below 0.05 denotes a test parameter’s significance at the 5% level, and multiple model variables were significant (*p* < 0.05). The model’s effectiveness and capacity to predict the percentage of dye decolorization were demonstrated by the similarity of adjusted R^2^ (0.8874) and predicted R^2^ (0.7641) and the difference between them didn’t exceed 0.2. The adequate precision of the model, which assesses signal to noise ratio, was 14.63, with a ratio greater than 4 is desirable and after excluding the non-significant factors (Table 5), the best mathematical model explaining the behavior of the process can be reduced to that presented in Eq. (2).

$$\begin{gathered} {\text{Decolorization of dye }}\left( \% \right) = - {73}.{6967} + 0.0{289958}*{\text{lignin conc}} + {4}.00{44}*{\text{tryptone conc}} + \hfill \\ {6}.{5}0{475}*{\text{pH}} + {3}.{49779}*{\text{incubation time}} + 0.00{237854}*{\text{lignin conc}}*{\text{tryptone conc}} + \hfill \\ 0.0{6965}0{2}*{\text{tryptone conc}}*{\text{incubation time}} + \left( { - 0.{121549}*{\text{pH}}*{\text{incubation time}}} \right) + ( - 0.{738434} \hfill \\ *{\text{tryptone conc}}^{2} ) + \left( { - 0.0{343354}*{\text{incubation time}}^{2} } \right) \hfill \\ \end{gathered}$$

**Table 4:** Analysis of variance (ANOVA) of AB decolorization percentage showing the effect of lignin concentration, tryptone concentration, pH, and incubation time (growth age before dye addition).

Source	Sum of squares	DF	Mean square	F-value	p-value	
Model	10,711.27	9	1190.14	24.64	< 0.0001	Significant
A-lignin conc	290.93	1	290.93	6.02	0.0245	
B-tryptone conc	1493.37	1	1493.37	30.92	< 0.0001	
C-pH	10.79	1	10.79	0.2233	0.6422	
D-incubation time	3765.30	1	3765.30	77.96	< 0.0001	
AB	8.15	1	8.15	0.1687	0.6861	
BD	402.37	1	402.37	8.33	0.0098	
CD	136.16	1	136.16	2.82	0.1104	
B^2^	1196.92	1	1196.92	24.78	< 0.0001	
D^2^	6033.54	1	6033.54	124.92	< 0.0001	
Residual	869.36	18	48.30			
Lack of fit	607.10	13	46.70	0.8904	0.6043	Not significant
Pure error	262.25	5	52.45			
Cor total	11,580.63	27

**Table 5:** Fit statistics as obtained from design expert software.

**Std. Dev**	6.95	**R**^**2**^	0.9249
**Mean**	38.91	**Adjusted R**^**2**^	0.8874
**C.V. %**	17.86	**Predicted R**^**2**^	0.7641
		**Adeq precision**	14.6347

Consequently, by resolving this equation, the optimum levels of lignin conc, tryptone conc, pH, and incubation time were 583 mg/L, 5.4 g/L, 9.8, and 40.6 h, respectively, with a predicted decolorization percentage of 66.85%.

**Fig. 8:**
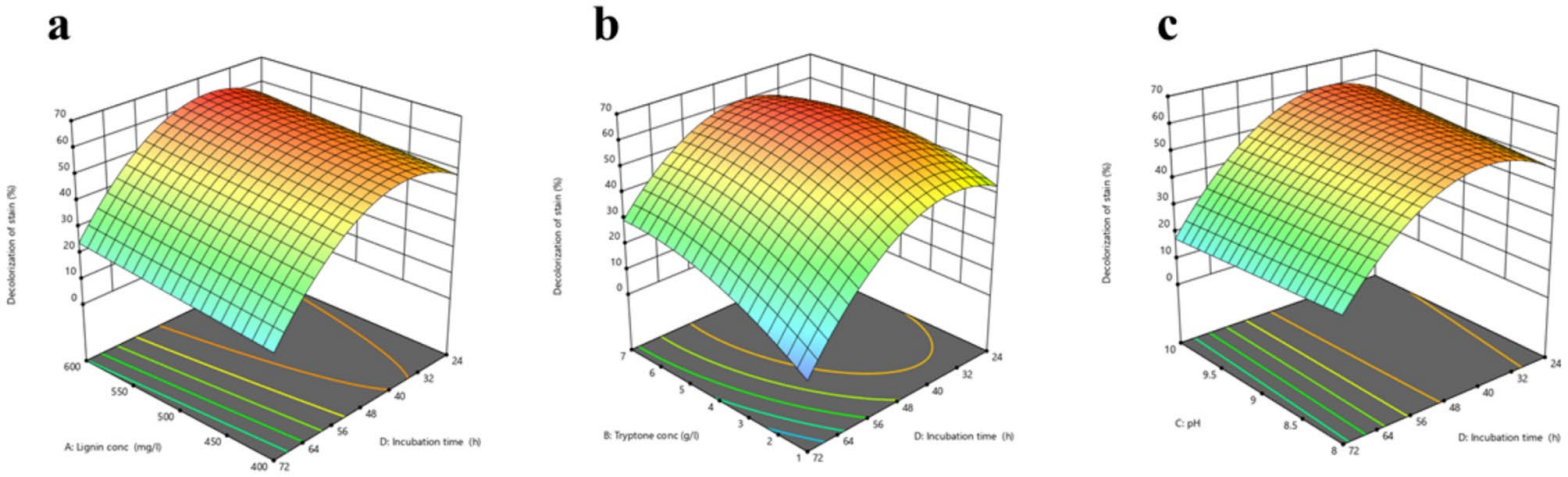
Three-dimensional (3D) surface plots for the effects of initial pH, lignin conc, tryptone conc and incubation time (growth age before dye addition) on AB decolorization percentage as obtained from design expert software.

The design-expert program was used to display a contour and response surface graph to investigate the relationship between the independent factors and the response. Figure 8a–c depicts these graphs, which demonstrate the quadratic effect of tryptone concentration and incubation time on the response. This effect can be explained by the statistical significance of the quadratic coefficients of these variables in the model with a convex shape in both axes (Fig. 8b). However, plots of pH and lignin content show a linear effect as seen in (Fig. 8a,c).

A statistically improved medium was used in two trials to confirm the validity of the experimental model. The response was 59.75 ± 6.94%, which indicated a strong resemblance between actual (59.75 ± 6.94%) and predicted values (66.85%) and confirming the accuracy and reliability of the model.

#### Dye decolorization in the basal and optimized conditions

**Fig. 9:**
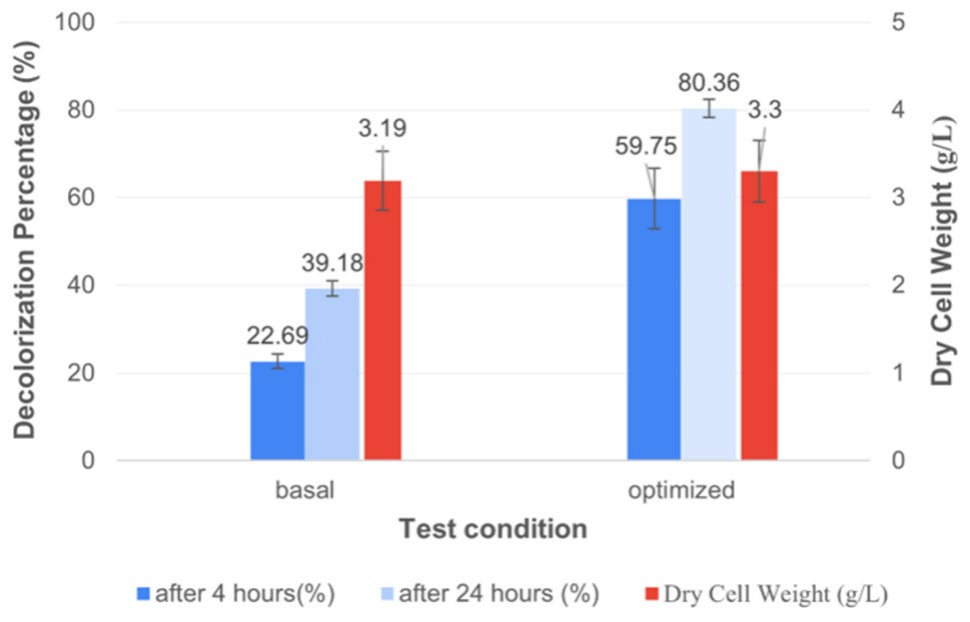
Decolorization percentage of AB in the basal and optimized conditions after 4 and 24 h of dye addition to the grown culture of *S. intermedius* test isolate. Lines above columns indicate standard deviation values.

Decolorization of AB dye (starting concentration of 6 mg/L) was accomplished using dye decolorization flasks from basal and optimized medium. Only 22.69% of the AB was decolorized from the basal medium after 4 h and 39.18% after 24 h contact periods after dye addition and re-incubation under the same conditions. While, by utilizing an optimized medium, it was observed that the corresponding decolorization percentage of the AB dye was increased to 59.75 and 80.36% after the same contact periods mentioned before (Fig. 9).

### Detection of the genes responsible for ligninolytic activity

#### Genomic DNA extraction of *S. intermedius* test isolate and quality control pretests

The DNA extraction from *S. intermedius* test isolate was performed, and the quality control pretests yielded the following outcomes, as presented in (Table 6 and Fig. S1). These outcomes indicate that the DNA extraction was successful, with a sample concentration of 37.2 ng/µL. Additionally, the sample integrity test indicated slight degradation. The overall assessment categorizes the sample as level A, signifying that it meets the necessary criteria for library construction and sequencing. The success rate of the sequencing process was around 93.59%.

**Table 6:** Quality control pretests on the genomic DNA of *S. intermedius* test isolate.

Sample name	Concentration (ng/µL)	Sample integrity	Library type	Test result
*S. intermedius isolate*	37.2	Degraded slightly	DNBSEQ WGS	Level A

Level A means the sample meets the requirements of library construction and sequencing. The current success rate is around 93.59%

#### Genome sequencing and assembly

In sequencing data, there is a certain number of low-quality reads in the raw data obtained. In order to obtain more accurate and reliable results in subsequent bioinformatics analysis, the raw data was treated by removing reads with a certain proportion of low quality, removing adapter contamination, and removing duplication contamination. The Q20%, Q30%, and GC% values were assessed for both pre-processed and post-processed data. Q20% increased slightly from 97.1 to 97.26%, while Q30% improved from 92.59 to 93.49%. The GC content remained relatively stable, changing from 72.44 to 72.1%. The above processes were applied to read 1 (forward read along the forward DNA strand), and read 2 (reverse read along the reverse DNA strand) of the whole genome sequence data obtained for *S. intermedius* test isolate and the statistical results are shown in (Table 7).

**Table 7:** DNBSEQ library statistics for the whole genome sequence data of *S. intermedius* test isolate.

Sample name	Insert size (bp)	Reads length (bp)	Raw data (Mb)	Adapter (%)	Duplication (%)	Total reads (#)	Filtered reads (%)	Low quality filtered reads (%)	Clean data (Mb)
*S. intermedius isolate*	350	(150:150)	1,241	0.50	0.04	8,274,060	9.09	8.03	1,128

*Insert size* the length of inserted fragment, *Reads length* length of reads, *Raw data* the size of raw data, *Adapter* the proportion of adapter, *Duplication* the proportion of same reads, *Total reads* total reads number, *Filtered reads* the proportion of filtered reads, *Low quality filtered reads* the proportion of low quality filtered reads, *Clean data* the size of delivered reads.

#### Bioinformatic analysis of whole genome sequence data of *S. intermedius* test isolate

The whole genome sequence obtained for *S. intermedius* test isolate after assembly was 7,084,373 bp in size, with 73.07% GC content, 6672 coding sequences (CDS) (Table 8), 4219 proteins with functional assignments, and 2453 hypothetical proteins (Table 9). According to morphological characteristics, 16S rRNA sequence, and complete genome sequence, this isolate was classified as a gram-positive bacterium, *Streptomyces* sp.

**Table 8:** Genomic features of *S. intermedius* test isolate as obtained from the sequence data.

Attribute	Value
Genome size (bp)	7,084,373
GC content	73.07
Contigs	139
Contig L50	14
Contig N50	155984
RNAs	73
Number of coding sequences	6672

**Table 9:** Protein features of *S. intermedius* test isolate as obtained from the sequence data.

Attribute	Value
Proteins with functional assignments	4219
Hypothetical proteins	2453
Proteins with Subsystem assignments	1272
Proteins with EC number assignments	1138

Genome sequence analysis of *S. intermedius* test isolate identified potential genes that may be accountable for degrading or depolymerizing lignin (Supplementary Materials, Table S1). After functional annotation of the genome sequence, the analysis showed the presence of bacterial peroxidases, especially dye decolorizing peroxidase (DyP) type. The genomic sequence of *S. intermedius* test isolate also contained a number of oxidoreductases, oxidases, reductases, and multicopper oxidases (laccases). Considering this, DyP and the laccase, which may be responsible for the enzymatic activity seen in this study may be brought on by the existence of these genes. Besides, it has been observed the presence of several genes encoding proteins that may be associated with the protection of cells against reactive species generated during aromatic compound metabolism. This includes superoxide dismutase, glutathione peroxidase, and thioredoxin genes. Additionally, it was found in the genome of this test organism genes related to the class of heme-thiolate proteins known as the cytochrome P450 genes.

Moreover, genome sequence analysis of *S. intermedius* test isolate identified the presence of multiple genes that are responsible for the catabolism of lignin derived compounds (Table S2). This includes the presence of multiple genes encoding enzymes responsible for aromatic funneling pathways such as hydroxybenzoate hydroxylase enzyme, vanillate demethylase, and dehydrogenases.

Additionally, the genes encoding enzymes involved in the metabolism of key aromatic intermediates through the ring cleavage pathway were discovered. These enzymes include catechol 1,2-dioxygenase, homogentisate 1,2-dioxygenase, 4-hydroxyphenylpyruvate dioxygenase, extradiol dioxygenase and multiple monooxygenases encoding genes (1,2-phenylacetyl-CoA epoxidase). Several transferase and hydratase encoding genes, including acetyl-CoA acetyltransferase and enoyl-CoA hydratase, which were previously reported to play a role in the breakdown of the aromatic molecule benzoate were also found. Additionally, the circular genome map that showed the distribution of some of the genes encoding ligninolytic enzymes was constructed using Proksee^49^ (Fig. S2).

As shown in Tables S1 & S2**,** the amino acid sequences of the *S. intermedius* test isolate’ genes that encode the lignin-degrading products were compared to other amino acid sequences that had been deposited in the NCBI database by using BLASTP algorithm to detect the similarity between them. By using this approach, we were able to locate the expected genes responsible for all of the pathways leading to lignin breakdown.

#### Submission of sequencing reads to NCBI

The FASTQ files of the WGS project of *S. intermedius* test isolate have been deposited in the NCBI/SRA database under the accession code SRR25321249.

## Discussion

It is approved that ligninolytic enzymes have a wide range of industrial uses. Besides, the production of numerous fine compounds from lignin and detoxification of pulp produced from paper industry processes^50^, they also have applications in versatile pollutant degradation such as insecticides, and polycyclic aromatic hydrocarbons (PAHs). They are helpful in cleaning up the environment since they may transform toxic substances into less dangerous forms^11^. Moreover, the ligninolytic enzymes produced by different microorganisms can also decolorize different types of dyes^25,51^. Those findings hold significant relevance in applying these enzymes in decolorizing textile dye effluent and bioremediation processes^21^. In this study, we could retrieve 177 potential ligninolytic bacteria from 55 soil samples using minimal salt medium containing KL. The use of lignin as the only source of carbon in minimal salt medium could encourage bacterial growth and raise the possibility that isolates may depolymerize lignin^52^. As mentioned in the literature, textile dye decolorization is one of the most significant uses of ligninolytic enzymes. Laccases and peroxidases, two types of ligninolytic enzymes, have been used to remove synthetic colors from textile effluent. By using them in textile processing, conventional treatments may be replaced with more environmentally friendly ones^11,53,54^. Therefore, assessing the isolates’ ligninolytic potential could be carried out through testing their capability to decolorize various dyes. Three different types of dyes were used in this study which included AB, MB, and CR. This method revealed that the bacterial isolate which demonstrated a unique capacity to decolorize distinct types of dyes with or without adsorption of the dye on its growth surface could have potential ligninolytic activity. Out of the total isolates tested, 16 of them displayed decolorization zones with the three different types of dyes used, indicating that they produce oxidative enzymes such as peroxidases and laccases which have the potential to act synergistically to depolymerize lignin. These isolates were subsequently selected for liquid phase testing to further explore isolates with the highest activity for potential application in lignin degradation and also for textile dye effluent bioremediation^21^. All tested isolates with decolorization activity on either tested dye displayed dye adsorption on cell pellets in the CR assay. Consequently, it is not clear to what extent isolates can degrade the dye rather than being adsorbed. Isolates having the code numbers 434 and 304 exhibited the highest activity by 98.5 and 72.5% in the AB assay and by 86.3% and 81% in the MB assay, respectively without adsorption of dye on cell pellets indicating that, among all the tested isolates, they have the strongest decolorizing activity. Based on the 16S rRNA sequence, isolates 434 and 304 are closely related to different *Streptomyces* groups, showing the highest similarity to *S. intermedius* strain DSM 40372 (NR_119347.1) and *S. griseorubens* strain NBRC 12780 (NR 041066.1), respectively. From the phylogenetic tree (Fig. 3), *S. rutgersensis* (NR_112495)^55^, *S. griseoflavus* (NR_042291), and *S. griseorubens* (NR_041066)^51^, *S. althioticus* (NR_115392)^56^ as well as *S. variabilis* (NR_043840)^57^ strains which are closely related to the two isolates used in this study, were also previously reported to demonstrate lignin-degrading ability. Dye decolorizing capacity in MSM-KL was greater than in LB medium indicating that lignin containing medium can induce the microorganism to produce ligninolytic enzymes within a shorter period of time. Several experiments have been carried out to determine factors affecting dye decolorizing activity by *S. intermedius* test isolate. The findings in this study showed that the activity was less affected by inoculum sizes (Fig. 4A). However, the maximum activity was observed within 48 h, and these results supported past research showing that actinomycetes exclusively generate ligninolytic enzymes as a growth-associated primary metabolic activity^58,59^. In this study, the decolorizing activity by *S. intermedius* isolate was maximum at 160 rpm. The same rpm was used for ligninolytic enzymes production by *Streptomyces* sp.S6^31^ compared to lower agitation (120 rpm)^60,61^ and higher agitation (200 rpm)^62^ used for such types of enzymes’ production. However, in this study, the increase in bacterial growth by agitation rates of 200 rpm, did not boost decolorization activity and consequently related degrading enzymes’ production. This could be interpreted on the basis that a high agitation rate increases the metabolic status of the microbial culture that might be balanced by a reflecting action demonstrated by enzymes synthesis lowering effect as a protective mechanism against cell lysis. Moreover, at lower agitation rates, enzyme output is reduced considerably which is probably attributed to a decrease in biosynthetic capability of the cells as a result of inadequate oxygenation and mixing of the culture media. The rate of agitation has a significant impact on how much dissolved oxygen is present in the fermentation medium^62^.

Additionally, it was observed that the optimum temperature was 30 ºC, a similar pattern of findings was reported in the biodegradation of KL by *Bacillus velezensis* strain^36^. Falade and his colleagues, also found that maximum LiP activity was obtained at a temperature of 30 °C and incubation time of 48 h^63^, which is likewise in good agreement with the optimum temperature (30 °C) and incubation time (48 h) reported in the present work. Most research articles on the biodegradation of lignin consistently report that the optimal temperature is 30 ºC^64^.

Some articles reported that ligninolytic bacteria can use KL as their only source of carbon without addition of extra carbon sources known as co-substrates^52,65^, while others reported that bacteria on fermentation media with glucose or the addition of another carbon source to the medium as a co-substrate can increase the production of ligninolytic enzymes^36,66^ or even is a must for stimulation of bacterial growth and co-metabolism of KL^67^. So, in this study, the effect of the addition extra carbon sources to MSM-KL was tested. The maximum activity was shown by no addition of extra carbon source to MSM-KL and thus validated that the isolate could utilize lignin as the only carbon source as a result of the lignin-inducing effect that drives the microorganism to manufacture lignin-degrading enzymes. The *S. intermedius* isolate was anticipated to depolymerize lignin and absorb the lower molecular weight KL-derived molecules to thrive^31^.

Based on the findings from previous studies, it has been observed that certain microorganisms exhibit limited tolerance to high concentrations of KL due to the tendency of KL to inhibit the growth of bacteria at higher concentrations^67,68^. To assess the tolerance effect of the isolate, varying concentrations of lignin were employed for testing this effect, the results revealed that *S. intermedius* isolate can tolerate a high concentration of lignin (1000 mg/L) with an optimum activity by using KL at a concentration of 600 mg/L which is higher than that found in pulp paper mill effluent (500 mg/L)^67^.

Enzyme production is influenced by the availability of nitrogen and its concentration in the media. The type of nitrogen supply may also affect the production and decolorization activity of ligninolytic enzymes^69^. The dye decolorization flasks were supplemented with several inorganic and organic nitrogen sources separately in an effort to identify an optimal nitrogen source for the decolorization activity. Regarding microbial growth, it was observed that some nitrogen sources had a significant effect on bacterial growth. The highest levels of decolorizing ability were observed when tryptone was used as a nitrogen source at a concentration of 0.4% w/v followed by peptone and yeast extract when used at the same concentration. On the contrary, urea and ammonium nitrate reduce microorganisms’ capacity to proliferate. DCW was significantly decreased when using urea and ammonium nitrate as a nitrogen source in comparison to other nitrogen sources used in this study, which may be the most likely reason for the decreased activity. This result ties well with previous studies wherein demonstrated that tryptone stimulates the highest laccase synthesis in comparison to yeast extract in *Bacillus* sp.^70^. Others found yeast extract as the optimum nitrogen source for the laccase production for the strain *S. psammoticus*^59,62^, while, Olajuyigbe and his colleagues^69^ reported that ammonium nitrate is the optimal nitrogen source for *Stenotrophomonas* sp for the production of ligninolytic enzymes.

The effect of initial pH on decolorization activity by *S. intermedius* test isolate was assessed by preparing fermentation media at different initial pH (6–12). The ranges of pH were selected according to previous studies of isolated ligninolytic bacteria^36^. Results obtained from this study showed that the optimum AB decolorization percentage from *S. intermedius* test isolate was achieved at pH 8.0 and 10. However, the decreased microbial growth and thereby enzymatic production at pH 12. These results revealed that pH is an important factor in decolorization activity. The results obtained in this study in agreement with previous studies reported that maximum ligninolytic enzyme production from *Bacillus* sp. SHC1 and *Leucobacter* sp.SHC3 occurred at pH 8^68^. It was also reported that *Cupriavidus basilensis* B-8, a bacterium with ligninolytic activity can grow at an alkaline pH range with optimum activity of KL degradation at pH 7^64^. However, most fungal isolate reports on the generation of ligninolytic enzymes indicated that production was best at an acidic pH^69,71^.

The level of the chosen variables was then optimized using RSM (CCD). During the evaluation of 3D response surface plots, it has been observed that tryptone and incubation time have an interactive effect on the percentage of dye decolorization with a convex shape in both axes (Fig. 8b) which means that the activity first increases gradually as tryptone and incubation time are increased to their peak levels, then decreases as tryptone levels are raised and incubation times are lengthened. The corresponding response surface plot’s convex form (Fig. 8b) suggested that the response’s optimal value falls within the range of the factors studied, a similar conclusion was reached by Aghaie-Khouzani^72^, who found that the maximum laccase activity falls within the optimum level of peptone concentration (2.2 g/L) upon using RSM. An alkaline pH range between 8 and 10 may boost the activity of ligninolytic enzyme production. These results go beyond previous reports, showing the same findings^36,63^. Consequently, when relevant components were used at their optimum levels, a 2.6-fold increase in the dye’s decolorization percentage was attained after 4 h of incubation with bacterium growth of *S. intermedius* isolate using the model’s optimal settings. These findings showed that the time of decolorization decreased, and the dye disappearance percentage increased, compared to unoptimized conditions.

The lignin’s intricate structure necessitates the cooperative action of several lignin-degrading enzymes that work in harmony. By applying WGS and annotation of genes, we were able to find and locate the anticipated genes in charge of all the processes leading to lignin degradation (Supplementary Materials, Tables S1, S2/Fig. S2). Two mechanisms are primarily responsible for lignin breakdown in nature: lignin is firstly depolymerized to produce low molecular weight lignin fragments, which are then either modified or biologically converted into other compounds. The depolymerization process of native lignin is induced by oxidative enzymes, such as peroxidases and laccases which finally leads to an oxidative degradation of bonds in lignin (Fig. S3A)^1^. The genomic sequence of *S. intermedius* isolate revealed the existence of multiple peroxidases and laccase genes. Peroxidase enzymes have been found to be the primary enzymes involved in lignin depolymerization^73^. DyP-type is a newly discovered family of peroxidases that has been reported to have broad substrate specificity including several types of dyes and lignin compounds^74,75^. Multicopper oxidase or laccase acts by reducing oxygen to water resulting in oxidation of phenolic and nonphenolic substances to their corresponding radical species which can then be subjected to further reactions^51,76^. It has been reported in several studies that the laccase enzyme produced by several *Streptomyces* sp can decolorize or degrade aromatic dyes^77^. So, the dye decolorizing activity observed in this study may be due to the presence of DyP and laccase encoding genes. As supporting enzymes, the oxidases produce hydrogen peroxides that the peroxidases use to break down lignin and aromatic compounds^78,79^. The oxidoreductase’s function is to produce unspecific free radicals and reactive intermediates in order to break down lignin and aromatic compounds^80^. These intermediates may be toxic and affect cell viability. Therefore, they must be eliminated or changed into safer and more stable molecules to allow microbial cell survival. Numerous ubiquinone oxidoreductase-encoding genes were found in *S. intermedius* isolate, which were tied with previous work by Jindal Li and his colleagues^81^ who reported the lignin degrading ability of this protein in *Aspergillus fumigatus*. Therefore, these genes may also be a sign of *S. intermedius* isolate’s capacity to degrade lignin. Several proteins, such as catalase, superoxide dismutase, glutathione peroxidase, and thioredoxin, associated with the oxidative stress response for defense against reactive species and detoxification pathways during aromatic metabolism, were also discovered in the genomic sequence of *S. intermedius* isolate. Genes encoded with cytochrome P450 proteins had also been found and were previously reported to assist lignin breakdown. Cytochrome P450 catalyzes many enzymatic reactions for the conversion of aromatic/xenobiotic compounds into more polar and/or less toxic derivatives^82^. When lignin is successfully depolymerized, a variety of substituted phenols and propyl phenols, as well as oligomers of these, are produced (Fig. S3A)^50^. After some modifications, those tiny fragments may be interesting chemicals on their own and represent one method of lignin valorization. The presence of several dehydrogenase encoding genes in the genomic sequence of *S. intermedius* isolate indicated that the isolate also had a role in the aromatic funneling pathway (Fig. S3B). Dehydrogenases act by transforming the various aldehydes produced into the equivalent carboxylic acid, which is typically less toxic to the host cell^1^. Different pathways were associated with the aromatic funneling process to produce central intermediate metabolites. The most common intermediate metabolite is protocatechuate. It has also been observed that the presence of two genes encoding enzymes of hydroxybenzoate hydroxylase and vanillate demethylase which are previously reported to have significant roles in the aromatic funneling process. Hydroxybenzoate hydroxylase acts by converting hydroxybenzoate into protocatechuate, vanillate demethylase acts by converting vanillic acid into protocatechuate^83^. This may ensure the potential role of this strain in the aromatic funneling pathways. The aromatic funneling pathways also produce some common central intermediates including catechol, protocatechuate, gentisate, and homogentisate, which will then go through central-ring cleavage catalyzed by ring-cleaving dioxygenase (Fig. S3B)^2^. Thus, the genomic analysis of *S. intermedius* isolate revealed the presence of another group of genes encoding enzymes that are responsible for romantic ring cleavages such as catechol dioxygenase, homogentisate dioxygenase, hydroxyphenylpyruvate dioxygenase, and monooxygenases, which contribute to the breakdown of central intermediate pathways. Some transferase and hydratase encoding genes were also detected in the genomic sequence of *S. intermedius* isolate. Those enzymes were also found to play a part in the breakdown of the aromatic molecule benzoate^84^. Finally, the analysis and identification of different metabolic pathways in its genomic sequence related to lignin degradation and dye decolorization activities indicated that we could consider this strain as a potential candidate for industrial application in lignin biodegradation and textile dye effluent bioremediation.

## Conclusion

The soil serves and can be considered as a reservoir for undiscovered or uncharacterized bacterial isolates that may be more beneficial in environmentally friendly processes. Upon isolation and characterization of bacterial isolates from various soil samples, *S. intermedius* test isolate 434 is beneficial as it used KL as a sole carbon source for growth and showed considerable decolorization efficiency of AB and other types of dyes. OFAT approach And RSM were used to maximize ligninolytic enzyme production and dye decolorizing capacity over a shorter period (4 h). This resulted in a 2.6-fold increase in the decolorizing capacity of AB within 4 h incubation with bacterium growth of the isolate. This study presents a novel approach to accelerating dye decolorization by optimizing ligninolytic enzyme production, offering a more efficient biotechnological solution.

Unlike previous studies, our work integrates optimization of dye decolorization with WGS, providing both functional and genetic insights into ligninolytic enzymes efficiency for industrial applications.

## Supplementary Information

Supplementary Information.

## Data Availability

All data generated or analyzed during this study are included in the manuscript and supplementary information files.

## Abbreviations

KL: Kraft lignin
MSM-KL: Mineral salt medium containing kraft lignin
LB: Luria–Bertani
AB: Azure B
MB: Methylene blue
CR: Congo red
DCW: Dry cell weight
WGS: Whole genome sequencing
LiP: Lignin peroxidase
MnP: Manganese peroxidase
VP: Versatile peroxidase
DyP: Dye decolorizing peroxidase
OFAT: One-factor-At-a-time approach
RSM: Response surface methodology
CCD: Central composite design
