# Supplementary material for: Screening and characterization of soil bacteria for lignin and textile dye effluent bioremediation and optimization using response surface methodology
Source: Sci Rep. 2025 Jul 16;15:25788. doi: 10.1038/s41598-025-04789-5 (PMC12267582; doi:10.1038/s41598-025-04789-5)
Supplement: Supplementary file 1 — Supplementary Information. [file 41598_2025_4789_MOESM1_ESM.docx]

**Table S1.** Annotated genes encoding lignin depolymerizing enzymes in S. intermedius test isolate

| **Feature ID** | **Function** | **Size (amino acid)** | **Organism** | **E-value** | **% Identity** | **Accession No.** |
| --- | --- | --- | --- | --- | --- | --- |
| **Peroxidases** | | | | | | |
| Iso 434.peg.1925 | Thiol peroxidase (EC 1.11.1.15) | 155 | [*Streptomyces*](https://www.ncbi.nlm.nih.gov/Taxonomy/Browser/wwwtax.cgi?id=1883) | 1e-108 | 100.00% | [WP_101277093.1](https://www.ncbi.nlm.nih.gov/protein/WP_101277093.1?report=genbank&log$=prottop&blast_rank=1&RID=B7K0CPGS013) |
| Iso 434.peg.2244 | Glutathione peroxidase (EC 1.11.1.9) | 162 | [*Streptomyces* sp. EAG2](https://www.ncbi.nlm.nih.gov/Taxonomy/Browser/wwwtax.cgi?id=2056495) | 2e-115 | 100.00% | [WP_101281052.1](https://www.ncbi.nlm.nih.gov/protein/WP_101281052.1?report=genbank&log$=prottop&blast_rank=1&RID=B7K0CPGS013) |
| Iso 434.peg.4209 | Putative Dyp-type peroxidase | 414 | [*Streptomyces*](https://www.ncbi.nlm.nih.gov/Taxonomy/Browser/wwwtax.cgi?id=1883) | 0.0 | 100.00% | WP_101277793.1 |
| Iso 434.peg.5089 | Thioredoxin | 113 | [Unclassified *Streptomyces*](https://www.ncbi.nlm.nih.gov/Taxonomy/Browser/wwwtax.cgi?id=2593676) | 7e-76 | 100.00% | [WP_101277843.1](https://www.ncbi.nlm.nih.gov/protein/WP_101277843.1?report=genbank&log$=prottop&blast_rank=1&RID=B7K0CPGS013) |
| Iso 434.peg.3180 | Thioredoxin | 122 | [*Streptomyces koyangensis*](https://www.ncbi.nlm.nih.gov/Taxonomy/Browser/wwwtax.cgi?id=188770) | 5e-77 | 100.00% | [WP_117348516.1](https://www.ncbi.nlm.nih.gov/protein/WP_117348516.1?report=genbank&log$=prottop&blast_rank=1&RID=B7K0CPGS013) |
| Iso 434.peg.1121 | Catalase (EC 1.11.1.6) | 698 | *Streptomyces* | 0.0 | 99.86% | [WP_101276441.1](https://www.ncbi.nlm.nih.gov/protein/WP_101276441.1?report=genbank&log$=prottop&blast_rank=1&RID=B7K0CPGS013) |
| Iso 434.peg.2222 | Catalase (EC 1.11.1.6) | 483 | *Streptomyces* | 0.0 | 100.00% | WP_101278333.1 |
| Iso 434.peg.2334 | Catalase (EC 1.11.1.6) | 485 | [*Streptomyces koyangensis*](https://www.ncbi.nlm.nih.gov/Taxonomy/Browser/wwwtax.cgi?id=188770) | 0.0 | 99.59% | WP_203216060.1 |
| Iso 434.peg.2911 | Catalase (EC 1.11.1.6) | 758 | *Streptomyces koyangensis* | 0.0 | 100.00% | WP_117348458.1 |
| Iso 434.peg.5169 | Catalase (EC 1.11.1.6) | 487 | [*Streptomyces* sp. EAG2](https://www.ncbi.nlm.nih.gov/Taxonomy/Browser/wwwtax.cgi?id=2056495) | 0.0 | 99.18% | WP_101279579.1 |
| Iso 434.peg.5731 | Catalase (EC 1.11.1.6) | 546 | [*Streptomyces*](https://www.ncbi.nlm.nih.gov/Taxonomy/Browser/wwwtax.cgi?id=1883) | 0.0 | 100.00% | WP_101279031.1 |
| **Oxidoreductases** | | | | | | |
| Iso 434.peg.2948 | NADH-ubiquinone oxidoreductase chain H (EC 1.6.5.3) | 294 | [*Streptomyces koyangensis*](https://www.ncbi.nlm.nih.gov/Taxonomy/Browser/wwwtax.cgi?id=188770) | 0.0 | 99.66% | WP_203216415.1 |
| Iso 434.peg.2950 | NADH-ubiquinone oxidoreductase chain K (EC 1.6.5.3) | 101 | [*Streptomyces* sp. EAG2](https://www.ncbi.nlm.nih.gov/Taxonomy/Browser/wwwtax.cgi?id=2056495) | 3e-61 | 100.00% | WP_101277736.1 |
| Iso 434.peg.2951 | NADH-ubiquinone oxidoreductase chain L (EC 1.6.5.3) | 610 | [*Streptomyces* sp. EAG2](https://www.ncbi.nlm.nih.gov/Taxonomy/Browser/wwwtax.cgi?id=2056495) | 0.0 | 99.84% | WP_101277735.1 |
| Iso 434.peg.2952 | NADH-ubiquinone oxidoreductase chain M (EC 1.6.5.3) | 502 | [*Streptomyces* sp. EAG2](https://www.ncbi.nlm.nih.gov/Taxonomy/Browser/wwwtax.cgi?id=2056495) | 0.0 | 99.20% | WP_101277734.1 |
| Iso 434.peg.2953 | NADH-ubiquinone oxidoreductase chain N (EC 1.6.5.3) | 471 | [*Streptomyces koyangensis*](https://www.ncbi.nlm.nih.gov/Taxonomy/Browser/wwwtax.cgi?id=188770) | 0.0 | 99.79% | WP_117348435.1 |
| Iso 434.peg.3812 | NADH-ubiquinone oxidoreductase chain D (EC 1.6.5.3) | 380 | *Streptomyces* | 0.0 | 100.00% | WP_030698886.1 |
| Iso 434.peg.5132 | FMN-dependent NADH-azoreductase (EC 1.7.1.6) | 220 | *Streptomyces* | 1e-153 | 99.55% | WP_101277869.1 |
| Iso 434.peg.5582 | NADH-ubiquinone oxidoreductase chain B (EC 1.6.5.3) | 216 | *Streptomyces* | 5e-154 | 100.00% | WP_101278686.1 |
| Iso 434.peg.5584 | NADH-ubiquinone oxidoreductase chain H (EC 1.6.5.3) | 322 | [*Streptomyces* sp. NBU3104](https://www.ncbi.nlm.nih.gov/Taxonomy/Browser/wwwtax.cgi?id=2911367) | 0.0 | 99.69% | WP_237896983.1 |
| Iso 434.peg.5585 | NADH-ubiquinone oxidoreductase chain I (EC 1.6.5.3) | 217 | [*Streptomyces koyangensis*](https://www.ncbi.nlm.nih.gov/Taxonomy/Browser/wwwtax.cgi?id=188770) | 8e-156 | 99.54% | WP_117349952.1 |
| Iso 434.peg.5586 | NADH-ubiquinone oxidoreductase chain J (EC 1.6.5.3) | 213 | *Streptomyces* | 4e-141 | 100.00% | WP_101278683.1 |
| Iso 434.peg.5587 | NADH-ubiquinone oxidoreductase chain K (EC 1.6.5.3) | 143 | *Streptomyces* | 5e-93 | 100.00% | WP_101278682.1 |
| Iso 434.peg.5588 | NADH-ubiquinone oxidoreductase chain L (EC 1.6.5.3) | 666 | *Streptomyces koyangensis* | 0.0 | 99.70% | WP_203214636.1 |
| Iso 434.peg.5589 | NADH-ubiquinone oxidoreductase chain M (EC 1.6.5.3) | 524 | *Streptomyces* | 0.0 | 100.00% | WP_101278680.1 |
| Iso 434.peg.5590 | NADH-ubiquinone oxidoreductase chain N (EC 1.6.5.3) | 513 | [*Streptomyces* sp. SCA2-2](https://www.ncbi.nlm.nih.gov/Taxonomy/Browser/wwwtax.cgi?id=1563677) | 0.0 | 99.61% | WP_129849507.1 |
| Iso 434.peg.6228 | NADH-ubiquinone oxidoreductase chain I (EC 1.6.5.3) | 232 | *Streptomyces* | 8e-171 | 100.00% | WP_101278714.1 |
| Iso 434.peg.6229 | NADH-ubiquinone oxidoreductase chain J (EC 1.6.5.3) | 271 | *Streptomyces* | 0.0 | 100.00% | WP_101278713.1 |
| Iso 434.peg.6230 | NADH-ubiquinone oxidoreductase chain K (EC 1.6.5.3) | 99 | *Streptomyces* | 1e-62 | 100.00% | WP_003948727.1 |
| Iso 434.peg.6231 | NADH-ubiquinone oxidoreductase chain L (EC 1.6.5.3) | 631 | *Streptomyces* | 0.0 | 99.84% | WP_203214665.1 |
| Iso 434.peg.6232 | NADH-ubiquinone oxidoreductase chain M (EC 1.6.5.3) | 533 | *Streptomyces* | 0.0 | 99.81% | WP_129849477.1 |
| Iso 434.peg.6233 | NADH-ubiquinone oxidoreductase chain N (EC 1.6.5.3) | 549 | *Streptomyces* | 0.0 | 99.82% | WP_101278710.1 |
| Iso 434.peg.6221 | NADH-ubiquinone oxidoreductase chain B (EC 1.6.5.3) | 184 | *Streptomyces* | 5e-133 | 100.00% | WP_100452179.1 |
| Iso 434.peg.6222 | NADH-ubiquinone oxidoreductase chain C (EC 1.6.5.3) | 251 | *Streptomyces* | 0.0 | 99.60% | WP_101278718.1 |
| Iso 434.peg.6223 | NADH-ubiquinone oxidoreductase chain D (EC 1.6.5.3) | 459 | *Streptomyces* | 0.0 | 100.00% | WP_101278717.1 |
| Iso 434.peg.6224 | NADH-ubiquinone oxidoreductase chain E (EC 1.6.5.3) | 291 | [*Streptomyces* sp. CZ24](https://www.ncbi.nlm.nih.gov/Taxonomy/Browser/wwwtax.cgi?id=2940554) | 0.0 | 99.66% | MDH6190756.1 |
| Iso 434.peg.6225 | NADH-ubiquinone oxidoreductase chain F (EC 1.6.5.3) | 449 | *Streptomyces* | 0.0 | 100.00% | [WP_030697618.1](https://www.ncbi.nlm.nih.gov/protein/WP_030697618.1?report=genbank&log$=prottop&blast_rank=4&RID=B7PBCY3M016) |
| Iso 434.peg.6226 | NADH-ubiquinone oxidoreductase chain G (EC 1.6.5.3) | 842 | [*Streptomyces koyangensis*](https://www.ncbi.nlm.nih.gov/Taxonomy/Browser/wwwtax.cgi?id=188770) | 0.0 | 100.00% | WP_117349935.1 |
| Iso 434.peg.6227 | NADH-ubiquinone oxidoreductase chain H (EC 1.6.5.3) | 458 | *Streptomyces* | 0.0 | 100.00% | [WP_101278715.1](https://www.ncbi.nlm.nih.gov/protein/WP_101278715.1?report=genbank&log$=prottop&blast_rank=1&RID=B7PBCY3M016) |
| **Superoxide dismutase** | | | | | | |
| Iso 434.peg.928 | Superoxide dismutase [Fe-Zn] (EC 1.15.1.1) | 212 | *Streptomyces* | 2e-154 | 100.00% | WP_101280481.1 |
| Iso 434.peg.2159 | Nickel-dependent superoxide dismutase (EC 1.15.1.1) | 131 | *Streptomyces* | 2e-91 | 100.00% | WP_003950798.1 |
| **Multicopper oxidase** | | | | | | |
| Iso 434.peg.1574 | Multicopper oxidase | 364 | [*Streptomyces koyangensis*](https://www.ncbi.nlm.nih.gov/Taxonomy/Browser/wwwtax.cgi?id=188770) | 0.0 | 100.00% | WP_117348640.1 |
| Iso 434.peg.2494 | Multicopper oxidase | 541 | [*Streptomyces koyangensis*](https://www.ncbi.nlm.nih.gov/Taxonomy/Browser/wwwtax.cgi?id=188770) | 0.0 | 99.63% | WP_203214179.1 |
| **Cytochrome P450** | | | | | | |
| Iso 434.peg.168 | Cytochrome P450 | 459 | *Streptomyces* | 0.0 | 99.78% | WP_101280205.1 |
| Iso 434.peg.2372 | Putative cytochrome P450 | 394 | [*Streptomyces* sp. MBRL 601](https://www.ncbi.nlm.nih.gov/Taxonomy/Browser/wwwtax.cgi?id=1592330) | 0.0 | 100.00% | KIX77285.1 |
| Iso 434.peg.3442 | Putative cytochrome P450 | 415 | [*Streptomyces* sp. MBRL 601](https://www.ncbi.nlm.nih.gov/Taxonomy/Browser/wwwtax.cgi?id=1592330) | 0.0 | 99.52% | KIX78851.1 |
| Iso 434.peg.4356 | Putative cytochrome P450 | 390 | [*Streptomyces griseus*](https://www.ncbi.nlm.nih.gov/Taxonomy/Browser/wwwtax.cgi?id=1911) | 0.0 | 97.95% | WP_115069054.1 |
| Iso 434.peg.4784 | Putative cytochrome P450 | 441 | [*Streptomyces koyangensis*](https://www.ncbi.nlm.nih.gov/Taxonomy/Browser/wwwtax.cgi?id=188770) | 0.0 | 100.00% | WP_117349584.1 |
| Iso 434.peg.4917 | Putative cytochrome P450 | 399 | [*Streptomyces* sp. EAG2](https://www.ncbi.nlm.nih.gov/Taxonomy/Browser/wwwtax.cgi?id=2056495) | 0.0 | 100.00% | WP_101278828.1 |
| Iso 434.peg.6068 | Putative cytochrome P450 | 422 | *Streptomyces* | 0.0 | 99.53% | WP_129848388.1 |
| Iso 434.peg.6259 | Putative cytochrome P450 | 405 | *Streptomyces* | 0.0 | 99.75% | WP_129847673.1 |
| Iso 434.peg.6284 | Putative cytochrome P450 | 398 | Unclassified *Streptomyces* | 0.0 | 100.00% | WP_101279618.1 |
| Iso 434.peg.6411 | Putative cytochrome P450 | 392 | [*Streptomyces pratensis*](https://www.ncbi.nlm.nih.gov/Taxonomy/Browser/wwwtax.cgi?id=1169025) | 0.0 | 98.98% | MBD2834389.1 |
| **Oxidases** | | | | | | |
| Iso 434.peg.1182 | Cytochrome c oxidase polypeptide II (EC 1.9.3.1) | 321 | [*Streptomyces albidoflavus*](https://www.ncbi.nlm.nih.gov/Taxonomy/Browser/wwwtax.cgi?id=1886) | 0.0 | 99.69% | WP_095709487.1 |
| Iso 434.peg.1183 | Cytochrome c oxidase polypeptide I (EC 1.9.3.1) | 578 | *Streptomyces* | 0.0 | 100.00% | WP_101277471.1 |
| Iso 434.peg.1184 | Cytochrome c oxidase polypeptide IV (EC 1.9.3.1) | 132 | *Streptomyces* | 5e-89 | 100.00% | WP_129850187.1 |
| Iso 434.peg.1187 | Cytochrome c oxidase polypeptide III (EC 1.9.3.1) | 206 | *Streptomyces* | 4e-147 | 100.00% | WP_047141635.1 |
| Iso 434.peg.2354 | Cytochrome c oxidase polypeptide I (EC 1.9.3.1) | 568 | [*Streptomyces* sp. EAG2](https://www.ncbi.nlm.nih.gov/Taxonomy/Browser/wwwtax.cgi?id=2056495) | 0.0 | 100.00% | WP_101280327.1 |
| Iso 434.peg.3253 | Choline oxidase (EC 1.1.3.17) | 505 | [*Streptomyces koyangensis*](https://www.ncbi.nlm.nih.gov/Taxonomy/Browser/wwwtax.cgi?id=188770) | 0.0 | 100.00% | WP_117350022.1 |
| Iso 434.peg.3482 | Cytochrome c oxidase polypeptide I (EC 1.9.3.1) | 564 | [*Streptomyces*](https://www.ncbi.nlm.nih.gov/Taxonomy/Browser/wwwtax.cgi?id=1883) | 0.0 | 100.00% | WP_101281447.1 |
| Iso 434.peg.4224 | Cytochrome d ubiquinol oxidase subunit II (EC 1.10.3.-) | 333 | *Streptomyces* | 0.0 | 100.00% | WP_101277806.1 |
| Iso 434.peg.4225 | Cytochrome d ubiquinol oxidase subunit I (EC 1.10.3.-) | 502 | *Streptomyces* | 0.0 | 100.00% | WP_101277807.1 |
| **Reductases** | | | | | | |
| Iso 434.peg.1663 | Respiratory nitrate reductase alpha chain (EC 1.7.99.4) | 1231 | [*Streptomyces koyangensis*](https://www.ncbi.nlm.nih.gov/Taxonomy/Browser/wwwtax.cgi?id=188770) | 0.0 | 99.84% | WP_117348680.1 |
| Iso 434.peg.1664 | Respiratory nitrate reductase beta chain (EC 1.7.99.4) | 541 | [*Streptomyces* sp. EAG2](https://www.ncbi.nlm.nih.gov/Taxonomy/Browser/wwwtax.cgi?id=2056495) | 0.0 | 100.00% | WP_101281388.1 |
| Iso 434.peg.1665 | Respiratory nitrate reductase delta chain (EC 1.7.99.4) | 197 | [*Streptomyces*](https://www.ncbi.nlm.nih.gov/Taxonomy/Browser/wwwtax.cgi?id=1883) | 3e-136 | 99.49% | WP_030694847.1 |
| Iso 434.peg.1666 | Respiratory nitrate reductase gamma chain (EC 1.7.99.4) | 236 | *Streptomyces* | 4e-165 | 99.58% | WP_101281386.1 |
| Iso 434.peg.2256 | Sulfite reductase, actinobacterial type (EC 1.8.7.1) | 565 | [*Streptomyces koyangensis*](https://www.ncbi.nlm.nih.gov/Taxonomy/Browser/wwwtax.cgi?id=188770) | 0.0 | 99.82% | WP_117348762.1 |
| Iso 434.peg.2461 | Putative reductase | 366 | [*Streptomyces* sp. EAG2](https://www.ncbi.nlm.nih.gov/Taxonomy/Browser/wwwtax.cgi?id=2056495) | 0.0 | 98.63% | WP_223282015.1 |
| Iso 434.peg.2501 | NADPH-dependent F420 reductase | 238 | [*Streptomyces*](https://www.ncbi.nlm.nih.gov/Taxonomy/Browser/wwwtax.cgi?id=1883) | 6e-169 | 100.00% | WP_101278571.1 |
| Iso 434.peg.2941 | 2,4-dienoyl-CoA reductase [NADPH] (EC 1.3.1.34) | 676 | [*Streptomyces* sp. EAG2](https://www.ncbi.nlm.nih.gov/Taxonomy/Browser/wwwtax.cgi?id=2056495) | 0.0 | 99.85% | WP_101277744.1 |
| Iso 434.peg.5088 | Thioredoxin reductase (EC 1.8.1.9) | 323 | [*Streptomyces*](https://www.ncbi.nlm.nih.gov/Taxonomy/Browser/wwwtax.cgi?id=1883) | 0.0 | 100.00% | WP_101277842.1 |
| Iso 434.peg.5132 | FMN-dependent NADH-azoreductase (EC 1.7.1.6) | 220 | [*Streptomyces*](https://www.ncbi.nlm.nih.gov/Taxonomy/Browser/wwwtax.cgi?id=1883) | 1e-153 | 99.55% | WP_101277869.1 |
| Iso 434.peg.5870 | Enoyl-[acyl-carrier-protein] reductase [NADH] (EC 1.3.1.9) | 259 | *Streptomyces* | 0.0 | 100.00% | WP_087776676.1 |

**Table S2.** Annotated genes encoding enzymes for lignin-derived aromatics catabolism in S. intermedius test isolate

| **Feature ID** | **Function** | **Size (amino acid)** | **Organism** | **E-value** | **% Identity** | **Accession No.** |
| --- | --- | --- | --- | --- | --- | --- |
| **Dehydrogenases** | | | | | | |
| Iso 434.peg.102 | Acyl-CoA dehydrogenase | 404 | [*Streptomyces* sp. EAG2](https://www.ncbi.nlm.nih.gov/Taxonomy/Browser/wwwtax.cgi?id=2056495) | 0.0 | 100.00% | WP_101280317.1 |
| Iso 434.peg.224 | 3-hydroxybutyryl-CoA dehydrogenase (EC 1.1.1.157) | 286 | *Streptomyces* | 0.0 | 100.00% | WP_101279528.1 |
| Iso 434.peg.390 | Acyl-CoA dehydrogenase, short-chain specific (EC 1.3.8.1) | 390 | *Streptomyces* | 0.0 | 99.74% | WP_101277199.1 |
| Iso 434.peg.86 | putative alcohol dehydrogenase (zinc-binding) | 363 | [*Streptomyces koyangensis*](https://www.ncbi.nlm.nih.gov/Taxonomy/Browser/wwwtax.cgi?id=188770) | 0.0 | 100.00% | WP_117350515.1 |
| Iso 434.peg.1614 | Aldehyde dehydrogenase (EC 1.2.1.3) | 499 | *Streptomyces koyangensis* | 0.0 | 100.00% | WP_117348661.1 |
| Iso 434.peg.1707 | 3-hydroxyacyl-CoA dehydrogenase (EC 1.1.1.35) | 606 | [*Streptomyces* sp. MBRL 601](https://www.ncbi.nlm.nih.gov/Taxonomy/Browser/wwwtax.cgi?id=1592330) | 0.0 | 100.00% | KIX77041.1 |
| Iso 434.peg.1713 | Acyl-CoA dehydrogenase | 401 | [*Streptomyces koyangensis*](https://www.ncbi.nlm.nih.gov/Taxonomy/Browser/wwwtax.cgi?id=188770) | 0.0 | 100.00% | WP_117348694.1 |
| Iso 434.peg.1551 | Acyl-CoA dehydrogenase | 380 | [*Streptomyces* sp. SCA2-2](https://www.ncbi.nlm.nih.gov/Taxonomy/Browser/wwwtax.cgi?id=1563677) | 0.0 | 99.74% | WP_129847166.1 |
| Iso 434.peg.2150 | Alcohol dehydrogenase | 321 | [*Streptomyces*](https://www.ncbi.nlm.nih.gov/Taxonomy/Browser/wwwtax.cgi?id=1883) | 0.0 | 99.69% | WP_101279236.1 |
| Iso 434.peg.2054 | 3-hydroxyacyl-CoA dehydrogenase (EC 1.1.1.35) | 282 | [*Streptomyces*](https://www.ncbi.nlm.nih.gov/Taxonomy/Browser/wwwtax.cgi?id=1883) | 0.0 | 100.00% | WP_101278046.1 |
| Iso 434.peg.2412 | Acyl-CoA dehydrogenase | 544 | [*Streptomyces*](https://www.ncbi.nlm.nih.gov/Taxonomy/Browser/wwwtax.cgi?id=1883) | 0.0 | 99.82% | WP_101278445.1 |
| Iso 434.peg.2267 | Acyl-CoA dehydrogenase | 544 | [*Streptomyces*](https://www.ncbi.nlm.nih.gov/Taxonomy/Browser/wwwtax.cgi?id=1883) | 0.0 | 99.63% | WP_101281428.1 |
| Iso 434.peg.2830 | Acyl-CoA dehydrogenase | 408 | [*Streptomyces*](https://www.ncbi.nlm.nih.gov/Taxonomy/Browser/wwwtax.cgi?id=1883) | 0.0 | 100.00% | WP_101280694.1 |
| Iso 434.peg.2831 | Alcohol dehydrogenase (EC 1.1.1.1) | 337 | *Streptomyces* | 0.0 | 100.00% | WP_101280695.1 |
| Iso 434.peg.2964 | Alcohol dehydrogenase (EC 1.1.1.1) | 336 | [*Streptomyces koyangensis*](https://www.ncbi.nlm.nih.gov/Taxonomy/Browser/wwwtax.cgi?id=188770) | 0.0 | 100.00% | WP_117348430.1 |
| Iso 434.peg.3142 | 3-hydroxybutyrate dehydrogenase (EC 1.1.1.30) | 266 | *Streptomyces* | 0.0 | 100.00% | WP_101277931.1 |
| Iso 434.peg.3323 | Aldehyde dehydrogenase (EC 1.2.1.3) | 538 | *Streptomyces* | 0.0 | 100.00% | WP_101276815.1 |
| Iso 434.peg.4015 | Aldehyde dehydrogenase (EC 1.2.1.3) | 495 | [*Streptomyces* sp. KE1](https://www.ncbi.nlm.nih.gov/Taxonomy/Browser/wwwtax.cgi?id=1638939) | 0.0 | 99.60% | KLJ01844.1 |
| Iso 434.peg.4016 | Aldehyde dehydrogenase (EC 1.2.1.3) | 305 | [*Streptomyces*](https://www.ncbi.nlm.nih.gov/Taxonomy/Browser/wwwtax.cgi?id=1883) | 0.0 | 99.02% | WP_030304731.1 |
| Iso 434.peg.4255 | Phenylacetic acid degradation protein PaaN2 | 572 | [*Streptomyces koyangensis*](https://www.ncbi.nlm.nih.gov/Taxonomy/Browser/wwwtax.cgi?id=188770) | 0.0 | 99.48% | WP_203215347.1 |
| Iso 434.peg.4258 | 3-hydroxyacyl-CoA dehydrogenase (EC 1.1.1.35) | 294 | [*Streptomyces*](https://www.ncbi.nlm.nih.gov/Taxonomy/Browser/wwwtax.cgi?id=1883) | 0.0 | 100.00% | WP_101279761.1 |
| Iso 434.peg.4726 | 4-aminobutyraldehyde dehydrogenase (EC 1.2.1.19) | 481 | [*Streptomyces koyangensis*](https://www.ncbi.nlm.nih.gov/Taxonomy/Browser/wwwtax.cgi?id=188770) | 0.0 | 100.00% | WP_117348976.1 |
| Iso 434.peg.4733 | 4-aminobutyraldehyde dehydrogenase (EC 1.2.1.19) | 505 | [*Streptomyces*](https://www.ncbi.nlm.nih.gov/Taxonomy/Browser/wwwtax.cgi?id=1883) | 0.0 | 100.00% | WP_101280140.1 |
| Iso 434.peg.4746 | Aldehyde dehydrogenase | 481 | [*Streptomyces koyangensis*](https://www.ncbi.nlm.nih.gov/Taxonomy/Browser/wwwtax.cgi?id=188770) | 0.0 | 100.00% | WP_117348976.1 |
| Iso 434.peg.4896 | Acyl-CoA dehydrogenase | 386 | [*Streptomyces*](https://www.ncbi.nlm.nih.gov/Taxonomy/Browser/wwwtax.cgi?id=1883) | 0.0 | 99.74% | WP_101278846.1 |
| Iso 434.peg.4897 | 3-hydroxybutyryl-CoA dehydrogenase | 506 | [*Streptomyces* sp. EAG2](https://www.ncbi.nlm.nih.gov/Taxonomy/Browser/wwwtax.cgi?id=2056495) | 0.0 | 99.41% | WP_101278845.1 |
| Iso 434.peg.5337 | Acyl-CoA dehydrogenase | 385 | [*Streptomyces*](https://www.ncbi.nlm.nih.gov/Taxonomy/Browser/wwwtax.cgi?id=1883) | 0.0 | 100.00% | WP_101280971.1 |
| Iso 434.peg.5359 | Alcohol dehydrogenase (EC 1.1.1.1) | 360 | [*Streptomyces koyangensis*](https://www.ncbi.nlm.nih.gov/Taxonomy/Browser/wwwtax.cgi?id=188770) | 0.0 | 100.00% | WP_117350154.1 |
| Iso 434.peg.5448 | Alcohol dehydrogenase (EC 1.1.1.1) | 356 | *Streptomyces* sp. CZ24 | 0.0 | 98.03% | MDH6190013.1 |
| Iso 434.peg.5469 | Acyl-CoA dehydrogenase | 608 | *Streptomyces* | 0.0 | 100.00% | WP_101275824.1 |
| Iso 434.peg.5737 | Acyl-CoA dehydrogenase, short-chain specific (EC 1.3.8.1) | 368 | *Streptomyces* | 0.0 | 100.00% | WP_255246331.1 |
| **Dioxygenase** | | | | | | |
| Iso 434.peg.218 | 4-hydroxyphenylpyruvate dioxygenase (EC 1.13.11.27) | 354 | *Streptomyces* | 0.0 | 100.00% | WP_129850544.1 |
| Iso 434.peg.219 | Phytanoyl-CoA dioxygenase | 305 | *Streptomyces* | 0.0 | 100.00% | WP_101279532.1 |
| Iso 434.peg.81 | Homogentisate 1,2-dioxygenase (EC 1.13.11.5) | 437 | *Streptomyces* | 0.0 | 99.77% | WP_101281293.1 |
| Iso 434.peg.1056 | Tryptophan 2,3-dioxygenase (EC 1.13.11.11) | 239 | *Streptomyces* | 4e-173 | 100.00% | WP_101276543.1 |
| Iso 434.peg.1954 | 4-hydroxyphenylpyruvate dioxygenase (EC 1.13.11.27) | 387 | *Streptomyces* | 0.0 | 100.00% | WP_047470192.1 |
| Iso 434.peg.3003 | Extradiol ring-cleavage dioxygenase, class III enzyme, subunit B | 320 | [*Streptomyces* sp. EAG2](https://www.ncbi.nlm.nih.gov/Taxonomy/Browser/wwwtax.cgi?id=2056495) | 0.0 | 100.00% | WP_101277696.1 |
| Iso 434.peg.3015 | Homogentisate 1,2-dioxygenase (EC 1.13.11.5) | 400 | *Streptomyces* | 0.0 | 100.00% | WP_101277685.1 |
| Iso 434.peg.4367 | Gentisate 1,2-dioxygenase (EC 1.13.11.4) | 386 | [*Streptomyces albogriseolus*](https://www.ncbi.nlm.nih.gov/Taxonomy/Browser/wwwtax.cgi?id=1887) | 0.0 | 98.19% | MCP9990427.1 |
| Iso 434.peg.4804 | Tryptophan 2,3-dioxygenase (EC 1.13.11.11) | 297 | *Streptomyces* | 0.0 | 100.00% | WP_101276025.1 |
| Iso 434.peg.5682 | Catechol 1,2-dioxygenase 1 (EC 1.13.11.1) | 267 | [*Streptomyces koyangensis*](https://www.ncbi.nlm.nih.gov/Taxonomy/Browser/wwwtax.cgi?id=188770) | 0.0 | 99.25% | WP_117350090.1 |
| **Monooxygenase** | | | | | | |
| Iso 434.peg.4259 | 1,2-phenylacetyl-CoA epoxidase, subunit A (EC 1.14.13.149) | 326 | [*Streptomyces koyangensis*](https://www.ncbi.nlm.nih.gov/Taxonomy/Browser/wwwtax.cgi?id=188770) | 0.0 | 100.00% | WP_117349331.1 |
| Iso 434.peg.4260 | 1,2-phenylacetyl-CoA epoxidase, subunit B (EC 1.14.13.149) | 105 | *Streptomyces* | 1e-70 | 100.00% | WP_101279763.1 |
| Iso 434.peg.4261 | 1,2-phenylacetyl-CoA epoxidase, subunit C (EC 1.14.13.149) | 286 | [*Streptomyces* sp. EAG2](https://www.ncbi.nlm.nih.gov/Taxonomy/Browser/wwwtax.cgi?id=2056495) | 0.0 | 100.00% | WP_101279764.1 |
| Iso 434.peg.4262 | 1,2-phenylacetyl-CoA epoxidase, subunit D (EC 1.14.13.149) | 221 | [*Streptomyces*](https://www.ncbi.nlm.nih.gov/Taxonomy/Browser/wwwtax.cgi?id=1883) | 1e-149 | 100.00% | WP_101279806.1 |
| Iso 434.peg.4263 | 1,2-phenylacetyl-CoA epoxidase, subunit E (EC 1.14.13.149) | 373 | [*Streptomyces koyangensis*](https://www.ncbi.nlm.nih.gov/Taxonomy/Browser/wwwtax.cgi?id=188770) | 0.0 | 99.46% | WP_117349333.1 |
| **Hydratase** | | | | | | |
| Iso 434.peg.1535 | Enoyl-CoA hydratase (EC 4.2.1.17) | 255 | [*Streptomyces*](https://www.ncbi.nlm.nih.gov/Taxonomy/Browser/wwwtax.cgi?id=1883) | 1e-177 | 100.00% | WP_101278982.1 |
| Iso 434.peg.1801 | Enoyl-CoA hydratase (EC 4.2.1.17) | 266 | *Streptomyces koyangensis* | 0.0 | 100.00% | WP_117349154.1 |
| Iso 434.peg.4257 | Enoyl-CoA hydratase (EC 4.2.1.17) | 264 | [*Streptomyces* sp. EAG2](https://www.ncbi.nlm.nih.gov/Taxonomy/Browser/wwwtax.cgi?id=2056495) | 0.0 | 100.00% | WP_101279760.1 |
| Iso 434.peg.5019 | Enoyl-CoA hydratase (EC 4.2.1.17) | 255 | [*Streptomyces*](https://www.ncbi.nlm.nih.gov/Taxonomy/Browser/wwwtax.cgi?id=1883) | 3e-180 | 100.00% | WP_101278383.1 |
| Iso 434.peg.5898 | Enoyl-CoA hydratase (EC 4.2.1.17) | 266 | [*Streptomyces*](https://www.ncbi.nlm.nih.gov/Taxonomy/Browser/wwwtax.cgi?id=1883) | 0.0 | 100.00% | WP_101280238.1 |
| Iso 434.peg.5965 | Aconitate hydratase (EC 4.2.1.3) | 910 | [*Streptomyces* sp. EAG2](https://www.ncbi.nlm.nih.gov/Taxonomy/Browser/wwwtax.cgi?id=2056495) | 0.0 | 100.00% | PKR43105.1 |
| **Transferase** | | | | | | |
| Iso 434.peg.4361 | 3-oxoadipate CoA-transferase subunit B (EC 2.8.3.6) | 220 | [*Streptomyces albogriseolus*](https://www.ncbi.nlm.nih.gov/Taxonomy/Browser/wwwtax.cgi?id=1887) | 7e-154 | 97.73% | MCP9990422.1 |
| Iso 434.peg.4362 | 3-oxoadipate CoA-transferase subunit A (EC 2.8.3.6) | 230 | *Streptomyces albogriseolus* | 2e-158 | 98.26% | MCP9990423.1 |
| Iso 434.peg.4899 | 3-oxoadipate CoA-transferase subunit A (EC 2.8.3.6) | 316 | [*Streptomyces wadayamensis*](https://www.ncbi.nlm.nih.gov/Taxonomy/Browser/wwwtax.cgi?id=141454) | 0.0 | 99.68% | KDR63824.1 |
| Iso 434.peg.4898 | 3-oxoadipate CoA-transferase subunit B (EC 2.8.3.6) | 273 | [*Streptomyces*](https://www.ncbi.nlm.nih.gov/Taxonomy/Browser/wwwtax.cgi?id=1883) | 0.0 | 100.00% | WP_101278844.1 |
| Iso 434.peg.1297 | Histidinol-phosphate aminotransferase (EC 2.6.1.9) | 379 | *Streptomyces* | 0.0 | 99.74% | WP_101278105.1 |
| Iso 434.peg.4901 | Acetyl-CoA acetyltransferase (EC 2.3.1.9) | 412 | [*Streptomyces koyangensis*](https://www.ncbi.nlm.nih.gov/Taxonomy/Browser/wwwtax.cgi?id=188770) | 0.0 | 99.76% | WP_117348592.1 |
| **Other enzymes** | | | | | | |
| Iso 434.peg.4364 | Putative n-hydroxybenzoate hydroxylase | 422 | *Streptomyces griseus* | 0.0 | 95.97% | SUP59758.1 |
| Iso 434.peg.1604 | Vanillate O-demethylas | 350 | *Streptomyces* sp. EAG2 | 0.0 | 99.71% | WP_101279496.1 |
| Iso 434.peg.3013 | Fumarylacetoacetase (EC 3.7.1.2) | 401 | *Streptomyces* | 0.0 | 100.00% | WP_101277687.1 |
| Iso 434.peg.6235 | Fumarylacetoacetase (EC 3.7.1.2) | 406 | [*Streptomyces koyangensis*](https://www.ncbi.nlm.nih.gov/Taxonomy/Browser/wwwtax.cgi?id=188770) | 0.0 | 99.01% | WP_203214662.1 |

**
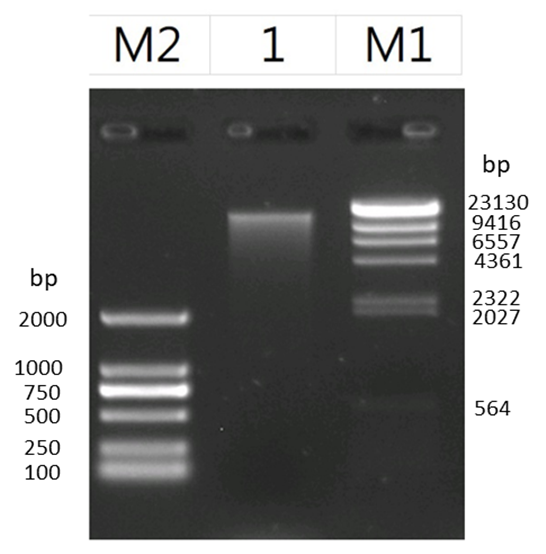
(A)**

**
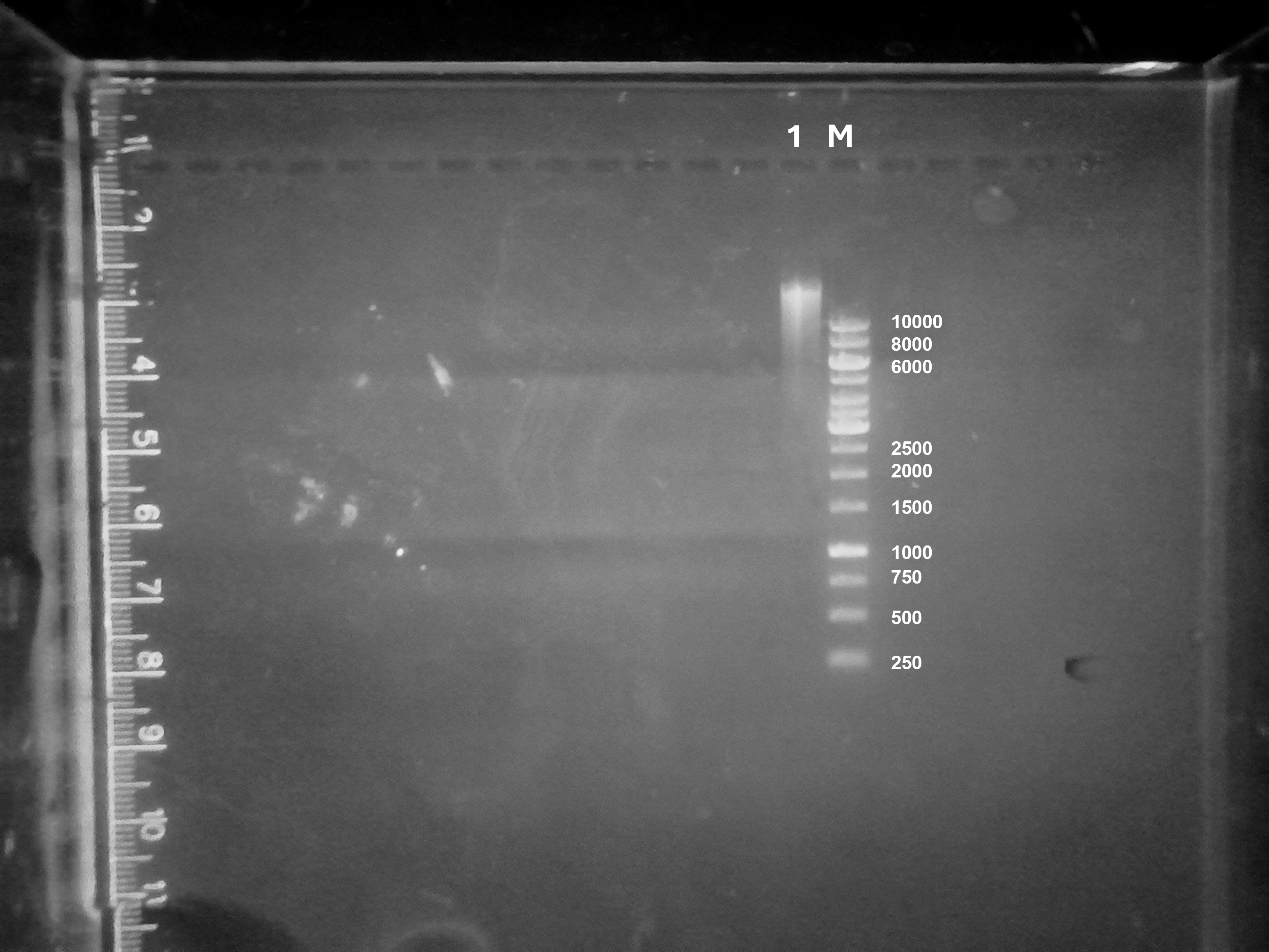
(B)**

**Fig. S1** Agarose gel electrophoresis of DNA extracted from *S. intermedius* test isolate. Lane M1; λ-Hind Ⅲ digest marker, Lane M2; D2000 marker and Lane 1; chromosomal DNA of *S. intermedius* test isolate as conducted by BGI Company **(A)**, and the same experiment conducted by the authors using different marker Lane M; GeneRuler 1 kb DNA ladder and Lane 1; chromosomal DNA of *S. intermedius* test isolate **(B)**.

**
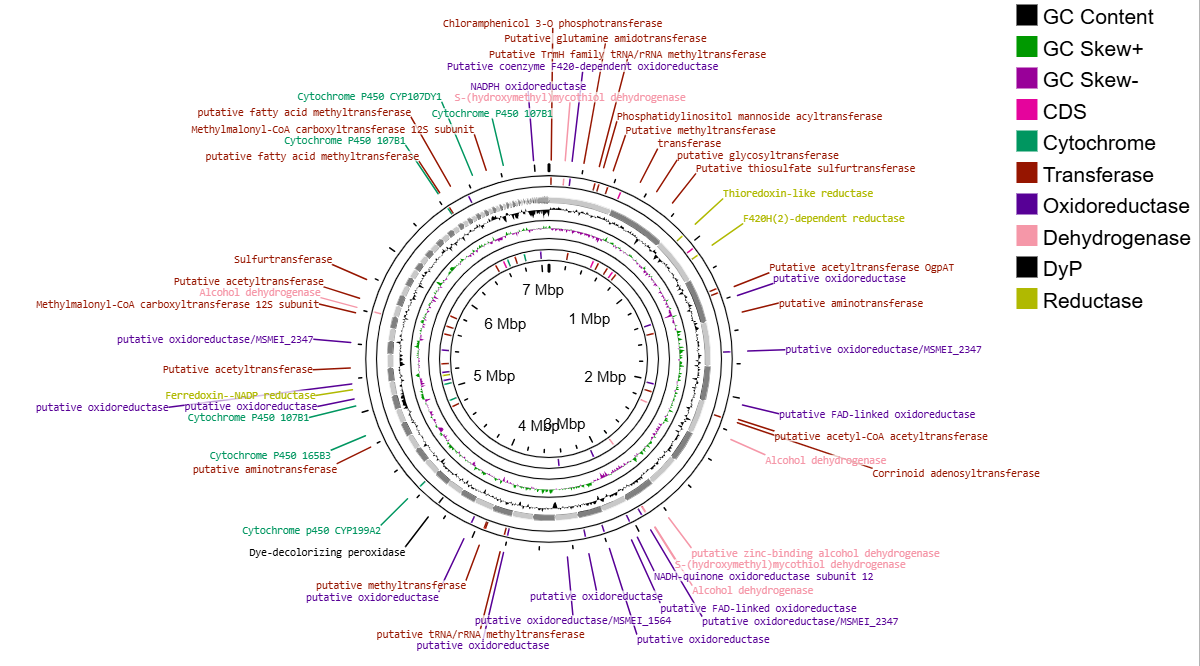
**

**Fig. S2** The circular map of the genomic sequence of Streptomyces isolate 434. The map highlights some selected genomic regions encoding enzymes potentially involved in lignin biodegradation. Gene locations are indicated on the circular layout, illustrating the spatial distribution of key ligninolytic genes within the genome. The figure was generated using Proksee (https://proksee.ca).

**(A)**


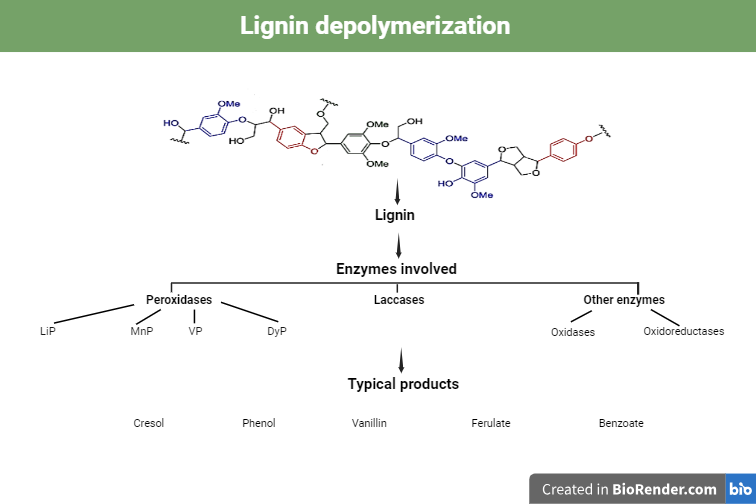


**(B)**


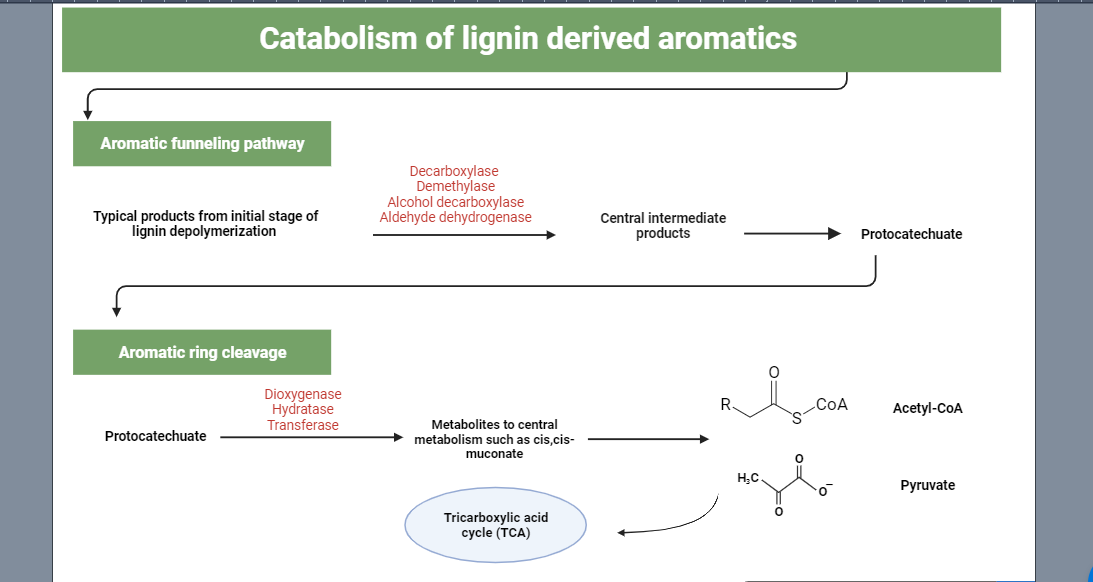


**Fig. S3** The most probably involved ligninolytic enzymes in the initial step of lignin degradation **(A)** and the second step of lignin breakdown (catabolism of the produced aromatics) **(B)**. LiP: lignin peroxidase, VP: versatile peroxidase, MnP: manganese peroxidase, DyP: dye decolorizing peroxidase (Milad et al., 2023).

.
